# Studying manganese carbonyl photochemistry in a permanently porous metal–organic framework†

**DOI:** 10.1039/d3sc03553k

**Published:** 2023-08-15

**Authors:** Rosemary J. Young, Michael T. Huxley, Lingjun Wu, Jack Hart, James O'Shea, Christian J. Doonan, Neil R. Champness, Christopher J. Sumby

**Affiliations:** a Department of Chemistry and the Centre for Advanced Nanomaterials, The University of Adelaide Adelaide Australia christopher.sumby@adelaide.edu.au; b School of Chemistry, The University of Nottingham Nottingham UK; c School of Chemistry, The University of Birmingham Birmingham UK n.champness@bham.ac.uk

## Abstract

Mn(diimine)(CO)_3_X (X = halide) complexes are critical components of chromophores, photo- and electrocatalysts, and photoactive CO-releasing molecules (photoCORMs). While these entities have been incorporated into metal–organic frameworks (MOFs), a detailed understanding of the photochemical and chemical processes that occur in a permanently porous support is lacking. Here we site-isolate and study the photochemistry of a Mn(diimine)(CO)_3_Br moiety anchored within a permanently porous MOF support, allowing for not only the photo-liberation of CO from the metal but also its escape from the MOF crystals. In addition, the high crystallinity and structural flexibility of the MOF allows crystallographic snapshots of the photolysis products to be obtained. We report these photo-crystallographic studies in the presence of coordinating solvents, THF and acetonitrile, showing the changing coordination environment of the Mn species as CO loss proceeds. Using time resolved experiments, we report complementary spectroscopic studies of the photolysis chemistry and characterize the final photolysis product as a possible Mn(ii) entity. These studies inform the chemistry that occurs in MOF-based photoCORMs and where these moieties are employed as catalysts.

## Abstract 2

Photo-liberation of CO from a Mn(diimine)(CO)_3_Br moiety anchored within a permanently porous MOF support is studied. The crystallinity and structural flexibility of the MOF allows crystallographic snapshots of the photolysis products to be obtained.

## Graphical abstract

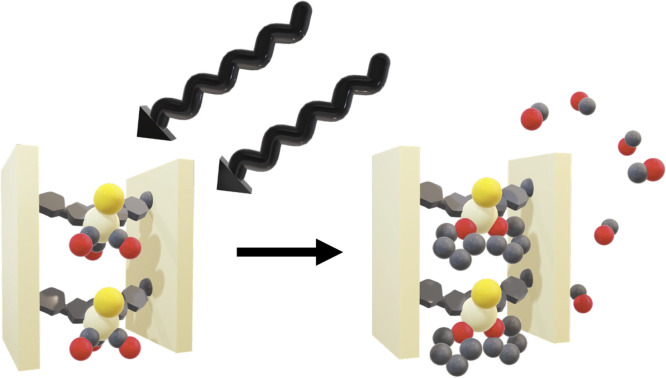

## Introduction

The Mn(diimine)(CO)_3_X (X = halide) family of complexes, and their Re analogues, have been widely studied^1^ as catalysts for both electro- and photocatalytic CO_2_ reduction,^2–6^ as light absorbing components in solar cells,^7^ biological probes^8,9^ and photoactive CO-releasing molecules (photo-CORMs).^10–12^ Central to the performance of the M(diimine)(CO)_3_X complexes in these processes is the ability to labilise, or retain CO, as required. For example, photocatalytic CO_2_ reduction necessitates the liberation of vacant coordinating sites to facilitate reaction of CO_2_,^5,13^ and while CO delivery by photo-CORMs is triggered by irradiation, an understanding of their stability and the timing and extent of CO release is needed to assess toxicity.^10^ Additionally, coordinatively unsaturated sites, generated by CO loss, possess interesting reactivity, including the possibility for CH activation and catalysis.^14^ Thus, further insight into the chemistry of these entities will expand the potential for new applications.

A strategy for studying photo-CORMs is to tether, or embed, the CO-bearing species within an isolating matrix such as a crystalline metal–organic framework (MOF).^15–18^ MOFs are periodic structures comprised of metal nodes connected by organic links^19^ that can form an open framework environment in which reactive species can be site isolated and studied. For example, we have used the flexible MOF, [Mn_3_L_2_L′] (MnMOF-1, where L = bis-(4-carboxyphenyl-3,5-dimethylpyrazolyl)methane) to study transformations of a Rh(i) dicarbonyl complex,^20^ observed site selective “click” reactions,^21^ and examined fundamental reaction processes,^22,23^ including for M(diimine)(CO)_3_X complexes.^24^ In these, and examples reported by others,^15,25–29^ the MOF structure acts as a rigid matrix preventing the recombination reactions of intermediate species. Furthermore, MOFs can provide a unique chemical environment that can be tailored to mimic the gas phase, or a solution phase, in which the quantity and type of solvent can be carefully controlled.^30^ Finally, MnMOF-1 in particular possesses a degree of structural flexibility that allows chemical reactions to occur without significantly damaging the crystal. This can provide definitive assessment of the localized structure of the metal centre by single crystal X-ray diffraction (SCXRD) before and after reaction.

**Fig. 1:**
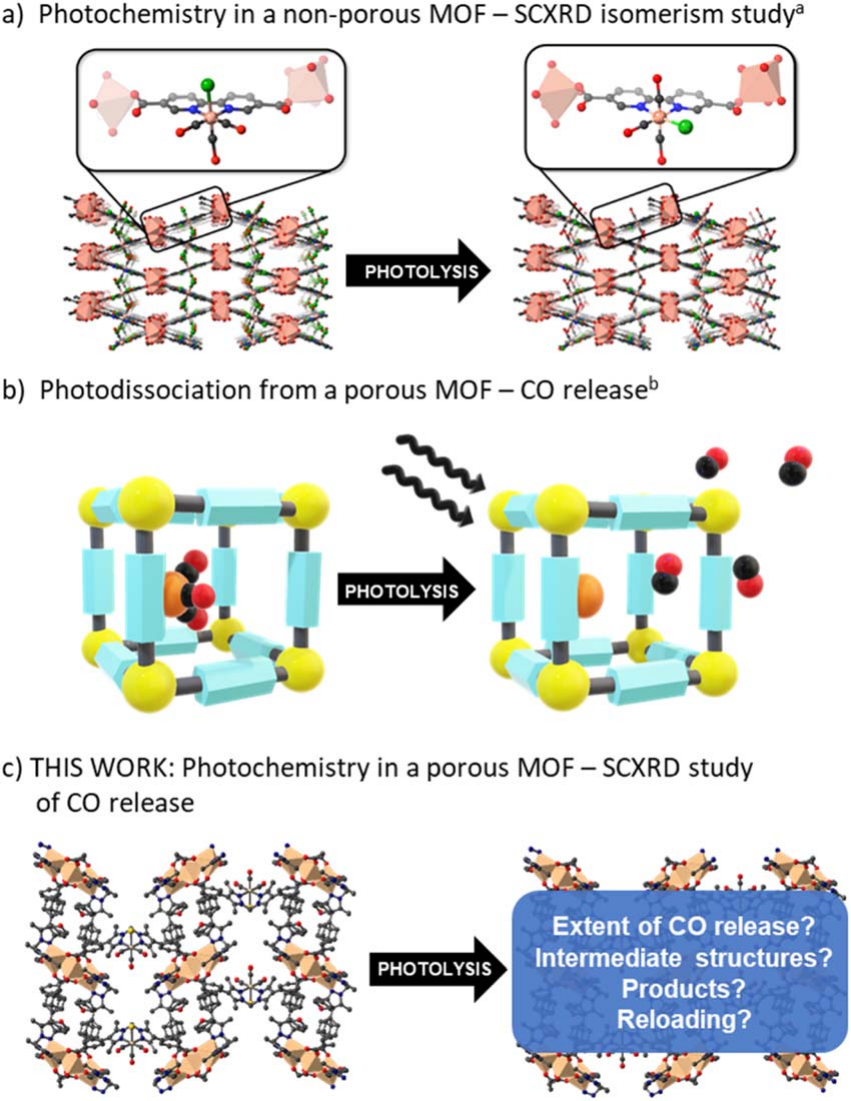
Representations of (a) prior art interrogating the photochemistry of M(diimine)(CO)_3_X in a MOF (note the Cl^−^ ligand shifts from an axial to equatorial site upon photolysis but is not quantitative and this leads to disorder) and (b) applying it to generate a MOF-based photoCORM. Herein, (c) we probe the chemistry of M(diimine)(CO)_3_X photolysis in a permanently porous MOF, providing crystallographic snapshots of the photolysis process.

Despite comprehensive studies of the photophysics and photochemistry of M(diimine)(CO)_3_X complexes being undertaken,^1,31,32^ how they behave supported within a solid-state matrix has only received limited attention. Blake *et al.* investigated the nature of the excited states and the formation of reactive intermediates for Re(diimine)(CO)_3_Cl and Mn(diimine)(CO)_3_X (X = Br, Cl) species by hosting the photoactive unit within a close-packed MOF (Fig. 1a).^33^ In the confined environment of this MOF (which is constructed from different Mn nodes), CO cannot be lost from the M(diimine)(CO)_3_X moiety and rearrangement from the *fac* to *mer* isomer was observed by SCXRD following photolysis and recombination. Follow-up contributions^34,35^ used spectroscopy to confirm the role of DMF solvent molecules trapped in the pores binding to the dicarbonyl intermediate and also examined the same photoactive unit in a MOF with a redox active node. In the latter example, photoinduced electron transfer was observed between the Re(diimine)(CO)_3_Cl unit and the Cu(ii) node.^35^

Studies of CO release from a Mn(diimine)(CO)_3_Br unit appended within an open porous framework have also been conducted.^36,37^ Diring *et al.* appended the same Mn carbonyl species within the pores of UiO-67-bpy (where bpy = 2,2′-bipyridine dicarboxylate) and showed light induced delivery and cellular uptake of CO, amounting to near quantitative release of framework sequestered CO (Fig. 1b).^36^ Further work in the same group used the Mn(diimine)(CO)_3_X units as nodes to produce amorphous mesoporous coordination polymers.^37^ Due to the small particle sizes of UiO-67-bpy and the amorphous nature of the coordination polymers, no definitive studies of the structure of the Mn intermediates generated during photolysis were reported. Owing to their importance as electro- and photocatalysts, Mn(diimine)(CO)_3_X units have been introduced into a range of other solid supports, including Periodic Mesoporous Organosilicas (PMOs).^38^ In these environments, spectroscopic detection of the *mer* isomer and a radical anion species, [Mn^+^(bpyPMO˙^−^)(CO)_3_] was made possible by persistent site-isolation, a property shared with MOFs. Again, due to a lack of long-range structural order, no crystallographic structural data was reported.

Herein we use the flexible MOF platform provided by MnMOF-1 to site isolate and undertake photochemical investigations of a Mn(diimine)(CO)_3_Br moiety anchored within a MOF (Fig. 1c). The system studied here is permanently porous, allowing not only the photo-liberation of CO ligands but their escape from the MOF crystals in the presence of different pore-filling solvents. Moreover, the single crystal-to-single crystal (SCSC) transformations, enabled by the structural flexibility of the MOF support, allows crystallographic snapshots of the photolysis products to be obtained. We report these photo-crystallographic studies in the presence of weakly and moderately strong coordinating solvents, THF and acetonitrile, showing the changing coordination environment of the Mn species as CO loss proceeds. Allied with these experiments we report spectroscopic studies of the photolysis chemistry and characterize the final photolysis product as a Mn(ii) entity.

## Results and discussion

### Synthesis and structure of MnMOF-1·[Mn(CO)_3_Br]

**Fig. 2:**
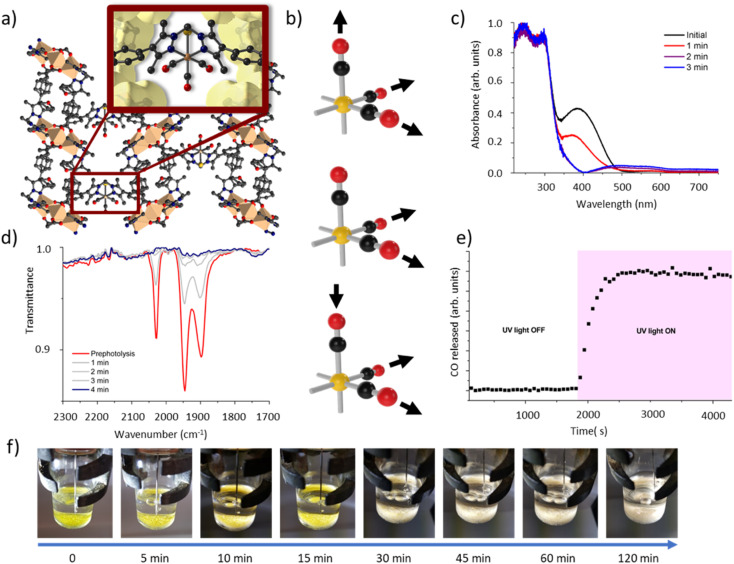
Details of the structure of MnMOF-1·[Mn(CO)_3_Br] and the bulk photolysis experiments. (a) The structure of MnMOF-1·[Mn(CO)_3_Br], highlighting the coordination environment of the Mn(CO)_3_Br entity, (b) the key IR modes, and (c) the solid-state UV-visible spectra of MnMOF-1·[Mn(CO)_3_Br] before and after photolysis. (d) IR spectra of the solid sample before and after photolysis and (e) bulk photolysis of a sample of MnMOF-1·[Mn(CO)_3_Br] measured by a residual gas analyser showing the CO is released from the MOF powder upon irradiation. (f) A visual representation of the photolysis process conducted in solvent under a N_2_ atmosphere. When crystals are photolysed in solution the CO release is notably retarded.

We have previously reported the quantitative metalation of MnMOF-1 with Mn(CO)_5_Br, to give MnMOF-1·[Mn(CO)_3_X]Y (X = solvent and Y = Br, or X = Br).^21,24^ The structure of MnMOF-1, formulated as [Mn_3_L_2_L′], is comprised of Mn(ii) trinuclear nodes coordinated to L (Fig. 2a), where in L all the donors are coordinated to the Mn framework nodes but L′ is coordinated solely through the carboxylate donors, thus providing a vacant bis-pyrazole site for metalation post-synthetically. This bis-pyrazole site is connected by a methylene hinge, which imparts remarkable flexibility while allowing maintenance of single crystallinity during post-synthetic metalation and subsequent chemical transformations. Formation of MnMOF-1·[Mn(CO)_3_Br] was achieved by treating the MOF with Mn(CO)_5_Br in ethanol at 50 °C to give MnMOF-1·[Mn(CO)_3_(EtOH)Br].^21^ This initial species is charge separated, with the bromide anion residing in the MOF pore and the Mn(i) ion coordinated to the bidentate MOF ligand, three carbonyl ligands and a solvent molecule. Exchanging the solvent for a comparatively poorly coordinating one such as THF converts this to the neutral complex, MnMOF-1·[Mn(CO)_3_Br], with the bromide anion replacing the solvent molecule.^24^

IR spectroscopy shed further light on this chemistry. The parent tricarbonyl complex MnMOF-1·[Mn(CO)_3_Br] exhibits three strong absorbance bands in the infrared, at approximately 2020, 1945, 1890 cm^−1^, with the positions of those bands varying slightly depending on the solvent and coordination environment (whether Br^−^ or solvent is coordinated).^24^ These absorbance bands correspond to the A′ and A′′ symmetric stretches and A′ asymmetric stretch of the *fac*-tricarbonyl complex (Fig. 2b).^39^ Irradiation of a THF solvated sample of MnMOF-1·[Mn(CO)_3_Br], dried under a flow of nitrogen gas to give a free flowing powder, with visible or UV light in an ambient atmosphere causes these bands to disappear, demonstrating that all three carbonyl ligands permanently dissociate from the Mn(i) centre under photolysis (Fig. 2d). Release of CO from a dried sample of MnMOF-1·[Mn(CO)_3_Br] under irradiation was also followed by residual gas analysis mass spectroscopy (RGA-MS), monitoring the *m*/*z* 28 peak (Fig. 2e). Initially, the sample was monitored in the dark to assess the stability of the carbonyl complex. No CO was detected at *m*/*z* 28 in the dark and so the sample was irradiated with UV light. An immediate, sharp increase in the *m*/*z* 28 peak was observed, which continued to increase for a period (13 min) before plateauing (the slow decrease over the remaining timescale of the experiment is due to sampling of the reaction headspace). Notably, no ongoing gradual increase in CO concentration was observed, as might be expected if CO gas trapped in MOF crystals was being slowly released. Thus, it can be concluded that the CO gas is not trapped in the MOF crystals but is released readily into the reaction cell.

### Matrix isolated IR spectroscopy of MnMOF-1·[Mn(CO)_3_Br]

**Fig. 3:**
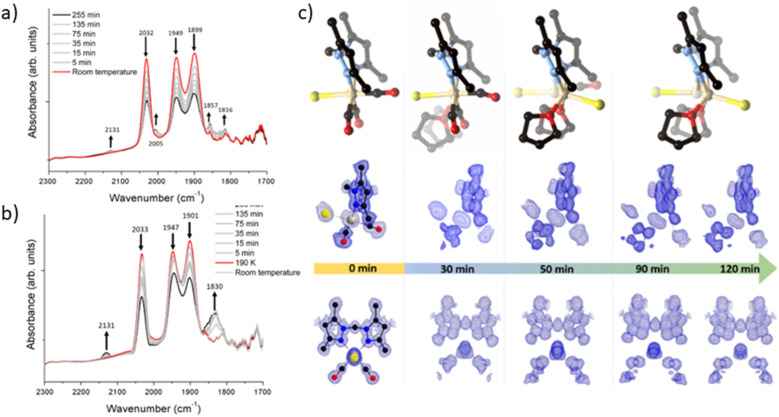
Details of the matrix isolated IR spectroscopy of MnMOF[Mn(CO)_3_Br] conducted at (a) room temperature and (b) 190 K showing the disappearance of the starting Mn(CO)_3_Br species and, in the experiment conducted at 190 K formation of a dicarbonyl intermediate. (c) A photocrystallography experiment conducted on MnMOF[Mn(CO)_3_Br] showing the modelled coordination environment that forms under photolysis and the associated electron density maps from individual data collections at 30, 50, 90 and 120 minutes.

To elucidate the steps involved in this photodissociation reaction, matrix isolated infrared spectroscopy was employed. In this technique the MOF sample, which was solvated with THF, was dried under a flow of nitrogen gas, and incorporated into a salt (NaCl or KBr) disk. The sample was placed in an IR cell under vacuum (approx. 10^−6^ mbar) and the photolysis reaction was then examined at room (296 K) and low temperature (190 K) (Fig. 3a and b). Photolysis with a xenon lamp (UV) at room temperature under vacuum elicited changes in the sample very similar to those observed for the bulk sample under ambient conditions, with the gradual disappearance of the three *fac*-tricarbonyl peaks. This process is much slower than that observed under ambient atmosphere or in the RGA experiment due to the retarding effects of the matrix. Notably, the appearance of new peaks at 1816, 1857 and 2005 cm^−1^ suggests the formation of new dicarbonyl and possibly monocarbonyl species. The peak that grows at 2131 cm^−1^ is due to CO gas being trapped in the matrix.^40,41^ Of more interest are the spectra collected at 190 K, which very clearly show the formation of a significant new peak at 1830 cm^−1^ (Fig. 3b), suggesting the formation of a single manganese di-carbonyl species. Kinetic analysis of the *fac*-tricarbonyl peaks reveal that the peak at 1947 cm^−1^ decreases at a slower rate than the other two peaks, indicating the presence of a second peak forming beneath it. The shifting of the new carbonyl stretches to lower wavenumbers indicates the formation of a complex with greater π back-bonding, consistent with the formation of dicarbonyl (and possibly monocarbonyl) complexes with an additional coordinated solvent molecule(s). This observation contrasts with a previously studied MOF system,^33^ where the close-packed nature of the structure favours *fac*–*mer* isomerisation and prevents CO loss.

### Photocrystallographic studies of MnMOF-1·[Mn(CO)_3_Br](THF) and MnMOF-1·[Mn(CO)_3_(MeCN)]Br

Given that the photolysis process proceeds *via* a series of intermediates, *in situ* photocrystallography experiments were undertaken to structurally characterise these species. These studies were conducted on a single crystal of the THF solvated sample MnMOF-1·[Mn(CO)_3_Br](THF), with multiple data collections on the same crystal following specified periods of irradiation. The experiment was conducted with irradiation from a LED light source (visible; as noted, the broad MLCT band is at 373 nm) at 270 K and data collections at 100 or 150 K, with the crystal slowly warmed and then cooled between each cycle of irradiation and collection. Initially the structure of the MOF-bound complex shows the *fac*-[Mn(CO)_3_Br] entity. Due to the asymmetry of the bis-pyrazole conformation, one axial site is more hindered than the other due to the proximity of the methylene bridge. In this case the Br^−^ ligand resides on the less hindered axial site, adjacent to the methylene bridge (Fig. 3c). After 30 min of irradiation, a change in the Mn coordination sphere is evident. The axial Br and CO ligands remain largely unaffected, but the equatorial sites are now disordered between CO and THF ligands, refined to *ca.* 50% occupancies. This is consistent with the matrix isolated IR results, which show that a dicarbonyl complex is initially formed at low temperature; here, the presence of a mirror plane means that the dicarbonyl species is disordered over both equatorial positions in the crystal structure. After 50 min of photolysis, further changes to the coordination sphere are evident with two THF molecules coordinated to the equatorial positions (occupancy fixed to that of the primary Mn centre). This change is accompanied by disorder of the Mn centre itself and the Br^−^ ligand over the two axial positions of the main Mn centre; these changes hinder further modelling of the structure. Electron density associated with the axial Br ligand also masks the presence, or absence, of a possible coordinated CO ligand. Disorder of the Br ligand over both axial positions at this step suggests some dissociation of the third CO axial ligand; however, whether it was released completely or recombined to form an isomer could not be confirmed. As photolysis progresses, this species may become a monocarbonyl complex or Mn complex without coordinated CO. Further photolysis did not modify the structure. It is notable that in the late-stage photolysis structures, the occupancy of the original manganese atom position decreases and the second position becomes more occupied (from *ca.* 60 : 40 at 50 min to 40 : 60 at 120 min).

Suspecting that the steric bulk of THF might preclude formation of a proposed fully substituted MnMOF-1·[Mn(THF)_3_Br] photoproduct, we instead pursued photocrystallography experiments using acetonitrile solvated crystals. Acetonitrile (MeCN), in contrast to THF, has a higher dielectric constant (*ε* = 37.5 *vs. ε* = 7.58)^42^ and a greater coordinating ability; thus the initial manganese carbonyl complex is charge separated, with the anion residing in the pore of the MOF. The structure is similar to that observed for the analogous PhCN system.^24^ An MeCN molecule is coordinated in the axial position (in the site anti to the methylene group) of the Mn(i), giving an initial *fac*-Mn(CO)_3_(NCMe) arrangement. During photolysis of this sample, a stronger, broad spectrum UV light was used to accelerate the photocrystallographic experiments, and the crystal was not warmed and cooled between collections to minimise crystal damage during the experiment (data collected at 100 K). After 10 min of photolysis a similar intermediate was formed as for THF (ESI, Fig. S8†). The equatorial positions of this photogenerated species contained disordered carbonyl and acetonitrile ligands with the occupancies refined to 25% and 75% respectively. As with the previous THF containing example, the axial ligands initially remain unchanged (although some axial CO loss cannot be discounted). After 20 min of irradiation the equatorial positions were occupied by MeCN ligands with 100% occupancy. The two axial positions which were initially occupied by the third CO ligand and a coordinated MeCN showed that whilst the axial MeCN remains coordinated, the axial CO was partially replaced by bromide (modelled at 25% and 33% respectively), as the third CO is released and replaced by the free bromide anion from the pore. Attempts to model the axial substituents to give the expected overall 100% occupancy resulted in over-assignment of the electron density. As with the THF solvated analogue, further irradiation did not fully resolve the identity of the final photoproduct. The structure became too disordered to model and the electron density at the Mn centre decreased, suggesting significant disorder or partial dissociation of Mn from the bis-pyrazole binding site (see ESI Section S1† for details of a leaching experiment that shows this is minimal for the THF species but slightly more pronounced for the MeCN solvated complex). Despite these limitations, photocrystallography experiments, where the photolysis is conducted *in situ*, allowed us to capture snapshots of key intermediates in the photo-dissociation reactions of Mn(CO)_3_X in the presence of two different solvents. However, the structural identity of the final species, observed by spectroscopy in the bulk phase experiments, and likely corresponding to the loss of all three carbonyl ligands, could not be determined due to poor crystal quality and disorder.

### Attempted identification of the final photoproduct

**Fig. 4:**
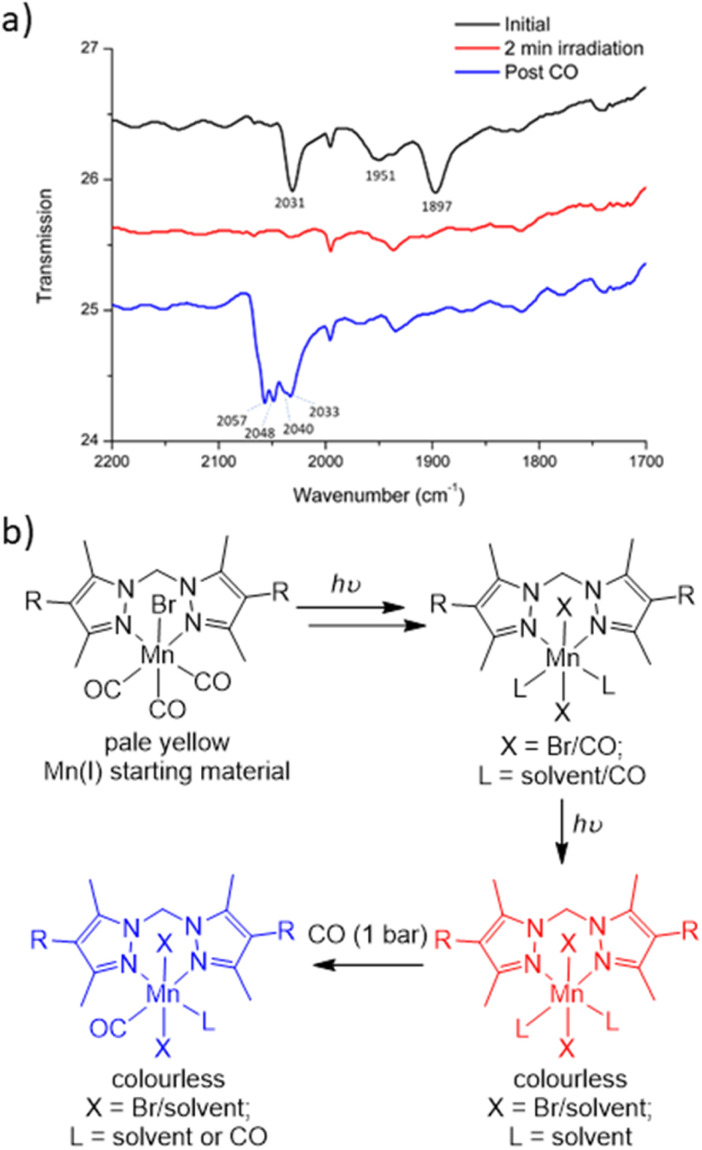
(a) Changes in coordination environment as monitored by changes in the CO stretching bands progressing from the starting material (black spectrum) to the CO loss product (red) and then giving the product (blue) with the CO stretches at higher wavenumbers, consistent with a Mn(ii) complex. The bands at *ca.* 2000 and 1935 cm^−1^ are present at all stages. (b) Proposed steps involved in CO photolysis of Mn(CO)_3_Br to give the colourless CO loss product (red) *via* intermediate di and monocarbonyl species. After photolysis, re-exposure of the sample to CO gives a proposed Mn(ii) species with one or more bound CO ligands (blue), possibly through introduction of an adventitious oxidant.

To characterise the product following loss of all three carbonyl ligands, a custom-made IR cell was built to perform the photolysis reaction under a controlled atmosphere.^43^ The photolysis of MnMOF-1·[Mn(CO)_3_Br] was performed under vacuum for 2 min and monitored by IR spectroscopy. Once the CO bands had disappeared, the cell was placed under a CO atmosphere at 1 bar. After 30 minutes of CO exposure, IR spectroscopy revealed the formation of new CO bands (2057, 2049, and 2041 cm^−1^) at higher wavenumbers than those of the parent complex (2027, 1951 and 1892 cm^−1^; Fig. 4a). This new manganese carbonyl complex failed to respond to irradiation with visible light, but irradiation with UV light (365 nm) caused bleaching of the new carbonyl bands. We considered a number of possibilities for the product, including Mn(CO)_5_Br (requiring displacement of the Mn from the NN binding site)^44^ or a potential bound [Mn(CO)_4_]Br^45^ complex, but both these species have a CO band above 2120 cm^−1^. We can also rule out a [Mn(CO)_3_(solvent)]Br species based on prior work.^24^

Given the shift in the CO bands to higher wavenumbers in this species, we tentatively postulated that a mixture of monocarbonyl or potentially dicarbonyl Mn(ii) photoproducts^46,47^ was being formed upon exposure to CO, with oxidation caused by an adventitious oxidant. The IR bands are consistent with a less electron rich metal centre.^1^ In an attempt to examine the potential oxidation state change, X-ray photoelectron spectroscopy (XPS) was employed to characterise the oxidation states of the Mn centre pre- and post-photolysis, as described in ESI Section S6.†^48^ The XPS data confirms that both MnMOF[Mn(CO)_3_X]Y pre- and post-photolysis samples contain both Mn(ii) and Mn(i) components. However, there was no measurable difference in the Mn spectra after photolysis, suggesting that in the vacuum environment of the XPS experiment (no adventitious oxidant) no oxidation state change occurred upon decarbonylation. A peak at *ca.* 635.5 eV was indicative of the presence of Mn(i) in both MOF samples (see ESI† for tabulated XPS data). Overall, the XPS data showed that the photolysis of MnMOF[Mn(CO)_3_X]Y under the inert, high vacuum conditions of the experiment does not lead to oxidation state changes for the Mn-species present.

To summarise the combined data (Fig. 4b), when photolysis of the MOF-bound Mn(CO)_3_Br moiety is undertaken in the presence or absence of solvent, stepwise CO loss occurs to give first a dicarbonyl species (loss of one equatorial CO), then the monocarbonyl species (loss of second equatorial CO), before all CO ligands are lost. Solvent clearly occupies both equatorial binding sites generated by CO loss but scrambling of the Br position and additional disorder prevents the identification of the expected sixth donor once the final CO ligand is lost. The resulting CO loss product can be treated with CO (1 bar) but does not recover the MnMOF[Mn(CO)_3_Br] starting material with, depending on the treatment, the Mn centre having appeared to undergo reaction with some adventitious oxidant due to the lack of stabilising CO ligands. Oxidation of Mn(i) photoCORMs to give a Mn(ii) species has been observed previously upon complete decarbonylation.^49^ Finally, the species formed from treatment of the photoproduct with CO, is resistant to CO photolysis under visible light but does bleach under UV irradiation.

## Conclusions

In this paper we demonstrate that there are significant differences in the photochemistry of a Mn(diimine)(CO)_3_X (X = halide or solvent) moiety when it is bound in a porous, as opposed to close-packed, MOF support; CO loss occurs in the former but in the close-packed structure isomerization occurs. While Mn(diimine)(CO)_3_X complexes have been widely studied as components of molecular and extended solid-state photoCORMs, limited insight into the steps and outcomes of the decarbonylation reactions has been provided. To achieve these insights, a highly crystalline MOF support, which can undergo complex single crystal-to-single crystal transformations, is critical. Here, taking advantage of such a platform, the photolysis of a Mn(diimine)(CO)_3_X moiety is shown to proceed *via* loss of the equatorial CO ligands, which in a solvated MOF crystal are progressively replaced by solvent molecules. Complete CO loss (from the remaining axial site) ensues, as shown spectroscopically, but this is accompanied by increasing disorder of the coordination environment that prevents ready characterization by SCXRD. It also appears, in the presence of adventitious oxidants but not an inert vacuum environment, the Mn(i) centre is oxidized to form a Mn(ii) complex, demonstrated by re-exposure to CO that leads to CO stretching bands at higher wavenumbers with respect to the starting complex and a very different response to visible light irradiation. This result has obvious implications for solid-state photoCORMs or use of these materials as safe CO generating materials, as the Mn(diimine)(CO)_3_X moiety cannot be regenerated. These observations also lead to questions about the stability of porous photocatalysts containing these Mn(diimine)(CO)_3_X moieties, which are typically exposed to UV and visible light for operation.

## Experimental

### General experimental

All chemicals were obtained from commercial sources and used as received unless otherwise stated. Dry solvents were distilled (acetonitrile over calcium hydride, THF over sodium/benzophenone and ethanol over magnesium) and degassed with argon before use. The ligand L (L = bis(4-carbonxyphenyl-3,5-dimethylpyrazolyl) methane),^50^ MnMOF-1,^20^ and solvent exchanged forms of MnMOF-1·[Mn(CO)_3_X]Y (where X = Br or, X = solvent and Y = Br)^21,24^ were prepared by previously reported methods. MnMOF-1·[Mn(CO)_3_X]Y was stored in the dark and handled with minimal exposure to light.

### Photolysis experiments and spectroscopy

Bulk photolysis was performed on 20–30 mg of sample in 5 mL of dry solvent, under constant flow of argon with an LEDlenser M14 400 lm visible torch. The sample and torch were wrapped in foil and the vial was agitated every 10 min to ensure even irradiation.

Solid state UV/vis spectra were obtained on a Cary 5000 UV/vis/NIR spectrophotometer with a Harrick Praying Mantis diffuse reflectance spectroscopy attachment on samples distributed in KBr.

Room temperature infrared (IR) spectra were collected on a PerkinElmer Spectrum Two spectrophotometer, with the sample dispersed between two NaCl disks inside a custom built sealed stainless-steel cell with NaCl windows, which allows for the sample to be placed under vacuum or a pressure of gas (see ESI† for further details). Samples were placed under vacuum for analysis. Samples exposed to CO were placed under vacuum, then 1 bar CO added before placed under vacuum again for analysis.

CO release from a solid MnMOF sample before and during photolysis was monitored using a small volume (3.26 cm^3^) stainless steel batch reactor fitted with a sapphire window for irradiation by a UV curing lamp. A pulse nozzle fitted to the cell is coupled to a residual gas analyser (RGA) which enables real time analysis of the reaction progression as a small portion of the head space is pulsed into the RGA at low pressure (2 × 10^−6^ Torr). The head space was monitored for a dried sample of MnMOF[Mn(CO)_3_Br] before and during irradiation.

Matrix isolated IR spectra were collected on a Thermo Nicolet Avatar 360 spectrophotometer with the sample dispersed in dry NaCl or KBr. MnMOF[Mn(CO)_3_X]. Y samples (2 mg) were incorporated into finely ground salt (KBr or NaCl) which had been dried in an oven at 100 °C for 2 days (200 mg), then pressed into a 9 × 0.5 mm disk under 6 tonnes of pressure in the dark. The disk was then placed into the sample chamber. The disk was placed into a copper cell with a lead gasket and CaF_2_ windows, which was sealed and placed inside a vacuum shroud, before being cooled to the analysis temperature. Photolysis was performed with a Philips HPK medium pressure 125 W mercury arc lamp.

### Single crystal X-ray diffraction (SCXRD)

Data for MnMOF[Mn(CO)_3_Br]·THF was collected on the MX1 beamline at the Australian Synchrotron^51^ and processed using XDS (*λ* = 0.7108).^52^ Single crystals were mounted in Paratone-N oil on a nylon loop. An initial structure was collected at 100 K, the crystal was then warmed to 270 K under N_2_ cryostream. A Ledlenser M14 visible LED torch was then positioned approx. 10 cm from the sample and turned on, the crystal was rotated periodically to ensure even irradiation. After 30 minutes of irradiation the sample was cooled to 150 K under irradiation, after-which the torch was removed, and a full sphere of data was collected. Following data collection, the crystal was warmed to 270 K and irradiated using the same procedure described above. This process of data-collection and irradiation was performed on the same single crystal for a total of 2 h of irradiation time, giving 4 structures at 30, 50, 90 and 120 minute irradiation times.

Data for MnMOF[Mn(CO)_3_MeCN]Br was collected on the i19 beamline at Diamond Light Source at 100K^53^ and processed using Xia2 software.^54^ Single crystals were mounted in Fomblin on a MiTeGen micromount. Irradiation was carried out at 100 K with a 3 W 365 nm LED in 10 min bursts.

*N*_tot_ reflections were merged to *N* unique (*R*_int_ quoted) after a multi-scan absorption correction and the structures were solved by direct methods using SHELXS^55,56^ or SHELXT^57^ and refined by full-matrix least-squares by SHELXL, interfaced through XSeed^58,59^ or Olex2.^60^ Unless stated otherwise, all non-hydrogen atoms were refined anisotropically and hydrogen atoms were included as invariants at geometrically estimated positions. The BYPASS routine in Olex2 ^61^ or the SQUEEZE routine of PLATON^62^ was used to subtract electron density corresponding to disordered solvent molecules where stated. Pertinent results are given in the text, while additional refinement details for each structure, thermal ellipsoid plots, and tables of crystallography data and refinement parameters are in the ESI.† CrystalMakerX and ShelXle were used to produce the figures. *F*_obs_ maps were produced at a resolution of 1.00 e per Å^3^.

### Powder X-ray diffraction (PXRD)

PXRD data was collected on a Bruker Advance D8 diffractometer equipped with a capillary stage and using Cu Kα radiation (*λ* = 1.5418 Å), or on a Bruker Endeavour D4 diffractometer using Co Kα radiation (*λ* = 1.789 Å).

### Scanning electron microscopy (SEM) and energy dispersive X-ray spectroscopy (EDX)

SEM/EDX was performed on a FEI Quanta 450 high resolution, field emission scanning electron microscopy with an Oxford Ultim Max large area SDD EDS detector or a Philips XL30 field emission scanning electron microscope with an Oxford X-Max SDD EDS detector. EDX spectra were processed with Oxford Aztec software. Data was collected on at least three crystals or areas of the SEM image.

### Manganese leaching experiment

MnMOF[Mn(CO)_3_Br]·THF [6 mg] was dried under argon, then photolyzed for 2 h under a flow of argon. Dry THF and CoCl_2_·10H_2_O (1 mg) was added and left overnight. The MOF was then washed with THF (×3), dried and analysed by SEM/EDX. The same procedure was followed for the acetonitrile solvated sample, MnMOF[Mn(CO)_3_MeCN]Br, with THF replaced by MeCN. Unphotolysed control samples were prepared by soaking the MnMOF[Mn(CO)_3_X]. Y (THF or MeCN solvated) (6 mg) with CoCl_2_·10H_2_O (1 mg) in the dark overnight, before washing them (×3) and drying them for EDX analysis.

### X-ray photoelectron spectroscopy (XPS)

Samples were prepared by drying to a free-flowing powder under a flow of N_2_ gas, then placed on an adhesive carbon tab on the sample holder. The sample was placed as quickly as possible into the Specs NAP-XPS instrument which was evacuated down to a pressure of 1 × 10^−9^ mbar. For all samples, low energy electron flood gun was used to neutralise any charging of the semi-insulating samples.

## Data availability

Information and data supporting this article have been uploaded as part of the ESI† and additional data is available from the authors on reasonable request. Crystallographic data have been deposited at the Cambridge Crystallographic Data Centre (CCDC 2219155–2219159 and 2219973–2219974).

## Author contributions

The project was conceived and supervised by CJS, NRC, and CJD. RJY and MTH contributed equally to the overall experimental aims of the study. The synthesis of materials and other experimental studies were conducted by RJY and MTH with help for specific experiments as follows. LW aided some IR spectroscopic experiments and JH and JO conducted XPS measurements. The initial draft of the manuscript was written by RJY, CJS and NRC. All authors discussed the results and contributed to the final preparation of the manuscript.

## Conflicts of interest

There are no conflicts to declare.

## Supplementary Material

SC-014-D3SC03553K-s001

SC-014-D3SC03553K-s002
