# Supplementary material for: Studying manganese carbonyl photochemistry in a permanently porous metal–organic framework
Source: Chem Sci. 2023 Aug 15;14(35):9409–17. doi: 10.1039/d3sc03553k (PMC10498678; doi:10.1039/d3sc03553k)
Supplement: SC-014-D3SC03553K-s001 [file SC-014-D3SC03553K-s001.pdf]

Electronic Supplementary Material (ESI) for Chemical Science.
This journal is © The Royal Society of Chemistry 2023

Supplementary Information for:

Studying manganese carbonyl photochemistry in a permanently porous Metal-organic Framework

Rosemary J. Young[a,b,†], Michael T. Huxley[a,†], Lingjun Wu[b], Jack Hart[b], James O'Shea[b], Christian J. Doonan[a], Neil R. Champness[b,c,*] and Christopher J. Sumby[a,*]

[a.] *Department of Chemistry and the Centre for Advanced Nanomaterials, The University of Adelaide, Adelaide, Australia. Email: christopher.sumby@adelaide.edu.au.*
[b.] *School of Chemistry, The University of Nottingham, Nottingham, UK.*
[c.] *School of Chemistry, The University of Birmingham, Birmingham, UK. Email: n.champness@bham.ac.uk.*

Table of Contents

Table of Contents 2

S1. Bulk characterisation and photo-induced leaching experiments for MnMOF-1·[Mn(CO)$_3$Br] (THF) and MnMOF-1·[Mn(CO)$_3$(MeCN)]Br 3

S2. Matrix isolated IR Spectroscopy 4

S2. FT-IR Spectroscopy 7

S3. Powder X-ray Diffraction (PXRD) Data 8

S4. Additional Single Crystal X-ray Diffraction (SCXRD) Structures, Refinement Details and Data 9

S4.1. Photocrystallography experiment for MnMOF-1·[Mn(CO)$_3$MeCN]Br 10

S4.2. Images of MnMOF-1·[Mn(CO)$_3$Br] (THF) pre- and post-photolysis 11

S4.3. Additional Refinement Details 12

4.4 Thermal ellipsoid plots of the asymmetric units for all structures 15

4.4.1 MnMOF-1·[Mn(CO)$_3$Br]·THF photocrystallography experiment 15

4.4.2 MnMOF-1·[Mn(CO)$_3$MeCN]Br photocrystallography experiment 20

S4.5. Tables of data collection and refinement parameters 22

S6. X-ray Photoelectron Spectroscopy (XPS) Details 26

S7. References 28

S1. Bulk characterisation and photo-induced leaching experiments for MnMOF-1·[Mn(CO)$_3$Br] (THF) and MnMOF-1·[Mn(CO)$_3$(MeCN)]Br

Table S1. Bromide anion occupancy for the MnMOF-1·[Mn(CO)$_3$Br] (THF) and MnMOF-1·[Mn(CO)$_3$(MeCN)]Br samples determined via measurement of the Mn:Br ratio using EDX analysis. Data is presented for pre- and post-photolysis samples to confirm that the counterion is not lost from the MOF pores.

**Sample**	Bromide occupancy (%)[a,b]	Std error (%)
MnMOF-1·[Mn(CO)$_3$(H$_2$O)]Br (As synthesised)	98	1.2
MnMOF-1·[Mn(CO)$_3$Br] (THF)	93	5.4
MnMOF-1·[Mn(CO)$_3$Br] (THF, soaked for 24hr after photolysis)[c]	92	5.0
MnMOF-1·[Mn(CO)$_3$(MeCN)]Br [c]	107	16

[a] Average atomic % obtained from three regions of crystals.

[b] Relative to the occupancy of the free bispyrazole coordination site in MnMOF-1.

[c] Following visible photolysis for three hours.

To investigate the small amount of possible leaching of the tethered Mn species seen during photo-crystallography experiments, a series of orthogonal metalation experiments were pursued. Bulk samples of MnMOF[Mn(CO)$_3$X].Y (THF and MeCN solvated) were dried under a stream of argon gas and photolyzed with visible light until no CO stretches were observable in the IR spectrum. As MnMOF-1 is readily metallated by CoCl$_2$, fully photolyzed crystals were placed under solvent (MeCN) and one molar equivalent of CoCl$_2$ added. The samples were left overnight, the solvent removed, and the crystals were analysed by Energy Dispersive X-ray analysis on a sample imaged by Scanning Electron Microscopy (Table S2). Samples which underwent the same procedure but without photolysis were analysed as controls.

In the case of the THF solvated sample, no significant additional replacement of the manganese with cobalt was observed compared to the control, suggesting limited leaching occurred under photolysis. In acetonitrile, slightly more significant replacement of Mn for Co was seen in the photolyzed sample compared to the control. The post-photolysis sample showed a 20% higher replacement, with over 50% of the manganese site replaced with cobalt (although the acetonitrile control also showed higher replacement compared to THF, indicating leaching is greater in acetonitrile even without photolysis). Overall, where photolysis experiments are conducted on solvated crystals leaching is an issue (e.g. in the photocrystallography), but this is minimised by the use of low temperature irradiation and data collection.

Table S2. Manganese leaching as determined by substitution with CoCl$_2$ and analysed by SEM/EDX.

Sample	Co occupancy	Standard Error
**THF control**	17%	5%
**Post-photolysis THF**	20%	4%
**MeCN control**	32%	3%
**Post-photolysis MeCN**	52%	3%

S2. Matrix isolated IR Spectroscopy

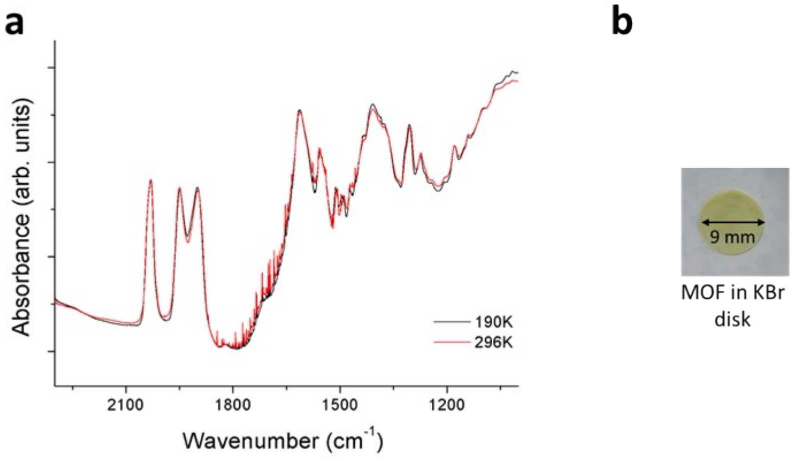

Figure S1. *a) Matrix isolated IR spectra of MnMOF-1·[Mn(CO)$_3$Br]·THF in a KBr disk at room temperature (296K, red) and cooled to 190K (black) and b) photo of MnMOF-1·[Mn(CO)$_3$Br]·THF pressed in a KBr disk.*

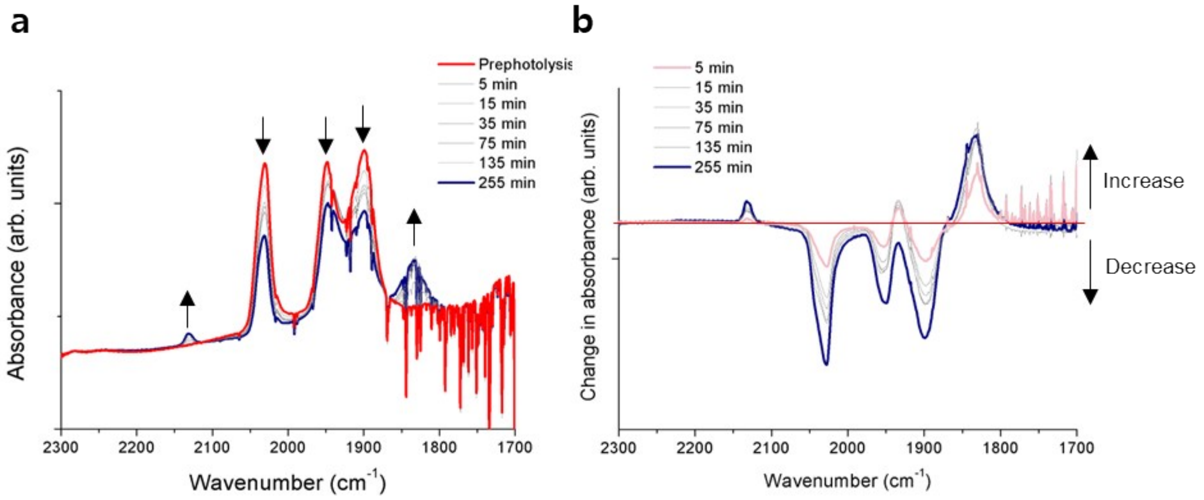

Figure S2*. a) Time resolved matrix isolated IR data for the carbonyl region during the photolysis of MnMOF-1·[Mn(CO)$_3$Br]·THF in a KBr disk at 190K. b) Difference spectra showing the change in absorbance between time points.*

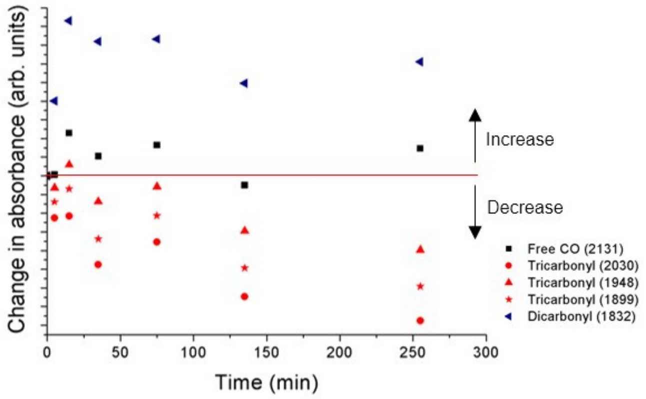

Figure S3. *Rate of change in absorbance of the carbonyl peaks of MnMOF-1·[Mn(CO)$_3$Br]·THF in KBr at 190 K during photolysis.*

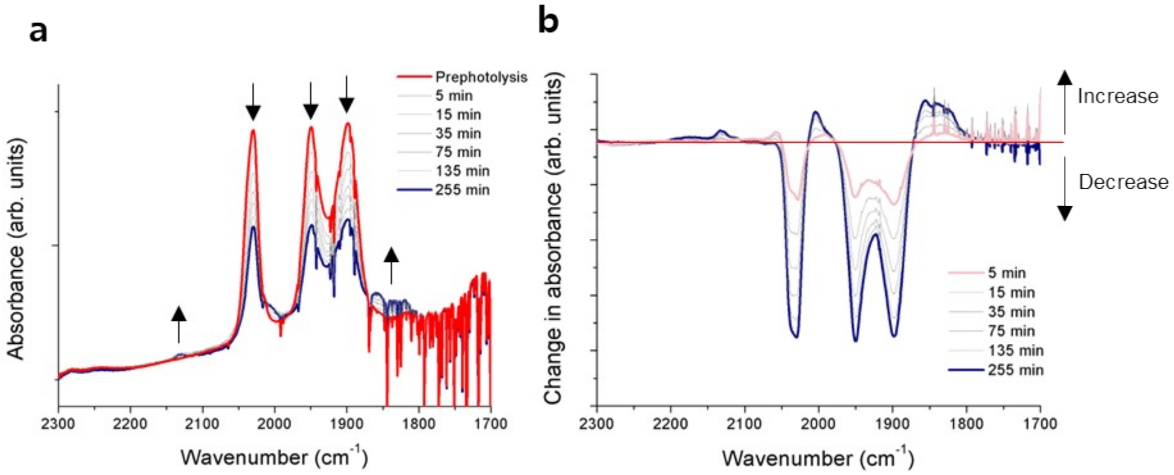

Figure S4. *a) Time resolved matrix isolated IR data for the carbonyl region during the photolysis of MnMOF-1·[Mn(CO)$_3$Br]·THF in a KBr disk at 296K. b) Difference spectra showing the change in absorbance between time points.*

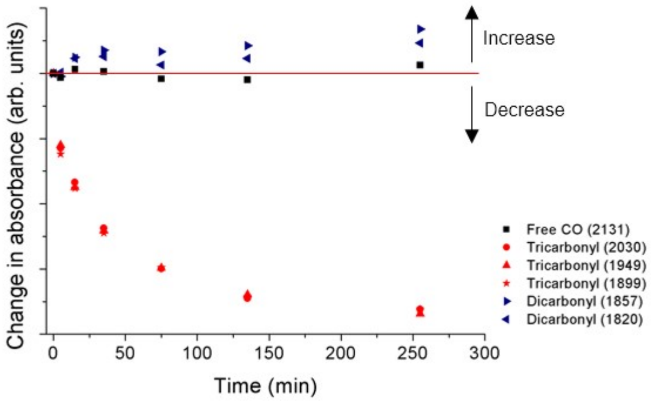

Figure S5. *Rate of change in absorbance of the carbonyl peaks of MnMOF-1·[Mn(CO)$_3$Br]·THF in KBr at 296 K during photolysis.*

S2. FT-IR Spectroscopy

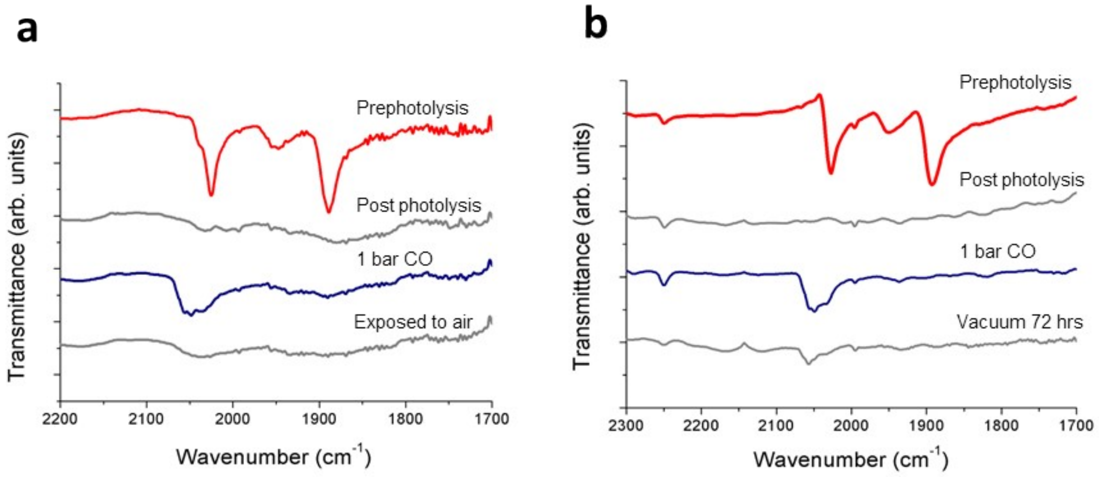

Figure S6. *MnMOF-1·[Mn(CO)$_3$X]Y samples photolysed with visible light in a controlled atmosphere IR cell then placed under CO gas. a) MnMOF-1·[Mn(CO)$_3$Br]·THF and b)MnMOF-1·[Mn(CO)$_3$MeCN]Br. Note that the intensity of the parent tricarbonyl band at 1951 cm^{-1} is weaker in intensity due to the particular instrument set-up.*

S3. Powder X-ray Diffraction (PXRD) Data

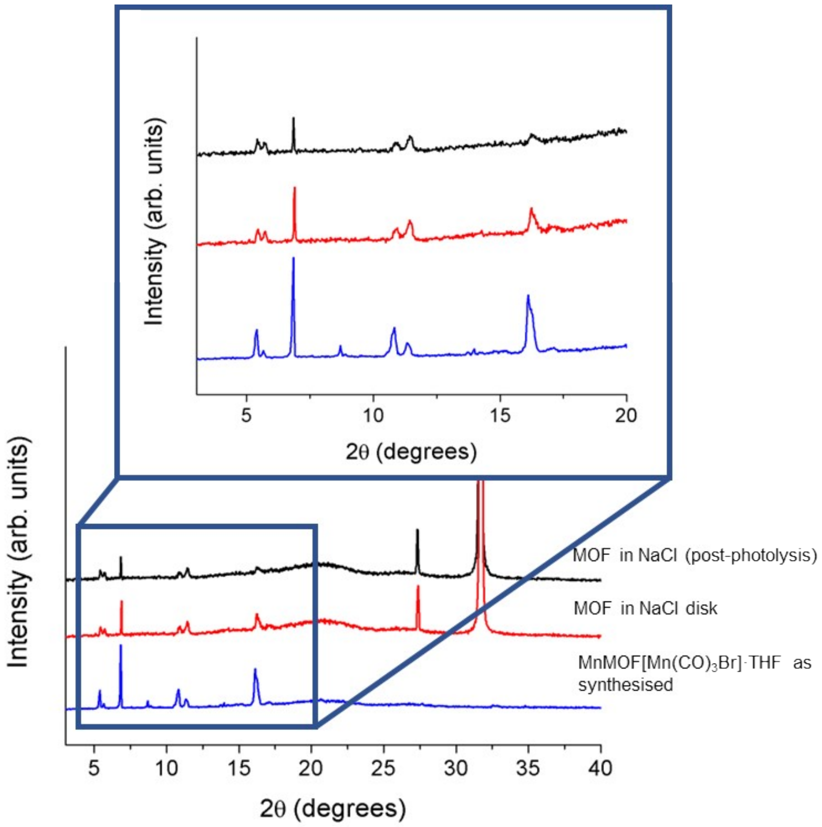

Figure S7. *PXRD data for MnMOF-1·[Mn(CO)$_3$Br]·THF as synthesised (blue), incorporated into an NaCl disk for matrix isolated IR studies (red) and in the NaCl disk after photolysis on the matrix isolated IR apparatus (black).*

S4. Additional Single Crystal X-ray Diffraction (SCXRD) Structures, Refinement Details and Data

Table S3. Summary of the structures, the data collection location and temperatures used for data collection and sample irradiation.

Sample	File name	CCDC Number	Data Collection Location	Data Collection temperature (K)	Irradiation temperature (K)
MnMOF-1·[Mn(CO)$_3$Br] THF	E1	2219155	Australian Synchrotron MX1 beamline	100	270
MnMOF-1·[Mn(CO)$_3$Br] (THF) (30 mins)	E130m	2219156	Australian Synchrotron MX1 beamline	150	270
MnMOF-1·[Mn(CO)$_3$Br] (THF) (50 mins)	E150m	2219159	Australian Synchrotron MX1 beamline	150	270
MnMOF-1·[Mn(CO)$_3$Br] (THF) (90 mins)	E190m	2219157	Australian Synchrotron MX1 beamline	100	270
MnMOF-1·[Mn(CO)$_3$Br] (THF) (120 mins)	E1120m	2219158	Australian Synchrotron MX1 beamline	150	270
MnMOF-1·[Mn(CO)$_3$MeCN]Br	2.3	2219974	Diamond Light Source i19 beamline	100	100
MnMOF-1·[Mn(CO)$_3$MeCN]Br (10 mins)	2.7	2219972	Diamond Light Source i19 beamline	100	100
MnMOF-1·[Mn(CO)$_3$MeCN]Br (20 mins)	2.8	2219973	Diamond Light Source i19 beamline	100	100

S4.1. Photocrystallography experiment for MnMOF-1·[Mn(CO)$_3$MeCN]Br

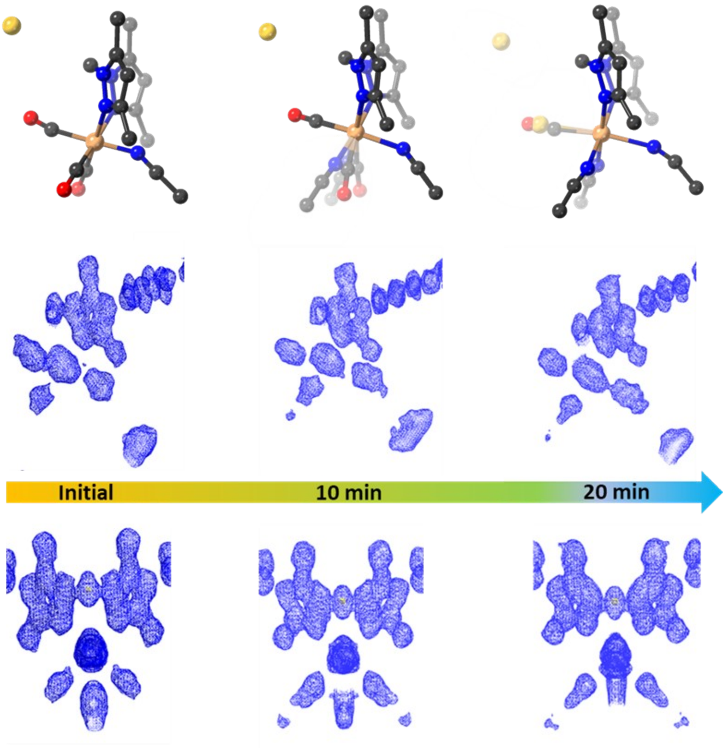

Figure S8. *Photodecarbonylation of a single crystal of MnMOF-1·[Mn(CO)$_3$MeCN]Br including F_{obs} maps with data collections taken* ***a)*** *pre-photolysis,* ***b)*** *after 10 min of irradiation and* ***c)*** *after 20 min of irradiation with UV light. The equatorial ligands in (b) (MeCN and CO) are disordered, with occupancies of 75% and 25% respectively. In (c) the occupancy of the Br^- in the pore is modelled at 33%, while the coordinated Br^- is at 25%. Occupancy of the axial carbonyl is fixed at 25%. (Mn, beige; C, grey; N, blue; O, red; Br, yellow).*

S4.2. Images of MnMOF-1·[Mn(CO)$_3$Br] (THF) pre- and post-photolysis

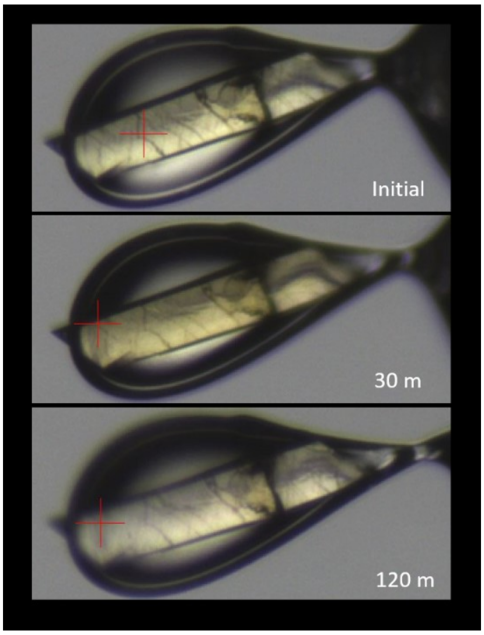

Figure S9. *Video microscope images of the single crystal of MnMOF-1·[Mn(CO)$_3$Br] (THF) used for the photocrystallography experiment. The grey background in the image makes colour differentiation difficult; however, it is evident that the crystal is initially yellow but becomes colourless after 120 minutes of photolysis consistent with UV-visible spectroscopy conducted on the bulk material.*

S4.3. Additional Refinement Details

MnMOF-1·[Mn(CO)$_3$Br] in THF (initial). The Mn occupancy of the individual crystal chosen for the photolysis experiment was determined to be 70% (0.35 site occupancy factor due to the mirror plane) by allowing the occupancy of the Mn and coordinated Br to refine. This Mn site occupancy was used for all subsequent photolysis experiments. The occupancy of the coordinated CO ligands was set on this basis. There is electron density in the “anion pocket” of the MOF and a small portion of Br (0.05 site occupancy factor) was modelled there; this implies that there is a small amount of solvent, likely water, coordinated to the Mn centre but due to the coordinated Br (0.30 site occupancy factor) this was not obvious in the difference map and not modelled. Rotational disorder of a phenyl moiety in the capping (pyrazole coordinated) ligand in the MOF was modelled by initially allowing the occupancy of the two positions (parts 1 and 2) to refine; the occupancy of the two positions was subsequently set at 60% and 40% respectively. There was also disorder of the phenyl moiety adjacent the·[Mn(CO)$_3$Br] metalation site but a satisfactory disorder model could not be refined. SIMU and RIGU restraints were included to help model the axially coordinated CO ligand and for the non-coordinated THF solvate molecule that is relatively well ordered in the pore (and retained in all subsequent structures). To subtract the contribution from the disordered solvent (THF; note, one THF molecule was located and refined), a solvent mask was calculated, and 396 electrons were found in a volume of 1342 Å^3 in 1 void per unit cell.[1] This is consistent with the presence of an additional 2.5[C4H8O] per Asymmetric Unit which account for 400 electrons per unit cell.

MnMOF-1·[Mn(CO)$_3$Br] in THF (30 min of visible photolysis). The occupancy of the Mn metalation site was refined and on the basis of this information, and the results from the unphotolysed sample, set to have be 70% (0.35 site occupancy factor). The Mn coordination sphere consists of an equatorial CO and THF ligand, disordered 50% over this site and axial Br and Br/CO ligands. The original axial CO site occupancy has dropped from a site occupancy factor of 0.35 to 0.30 and a small fraction of Br (0.05 site occupancy factor) appears in this site. The original coordinated Br site has dropped in occupancy from a site occupancy factor of 0.3 to 0.2 due to scrambling across the axial sites and an increase in the occupancy of the non-coordinated site to 0.1 (note, anion migration has been seen previously in crystals of this MOF).[2] DFIX restraints were used to restrain the Mn-Br, Mn-CO and C-O bond lengths to chemically reasonable values and SIMU/RIGU and ISOR restraints were used to stabilize the refinement of the coordination sphere. The coordinated THF was inserted as a fragment from the Idealized Molecular Geometry Library.[3] Rotational disorder of a phenyl moiety in the capping (pyrazole coordinated) ligand in the MOF was modelled by allowing the occupancy of the two positions (parts 1 and 2) to refine. There was also disorder of the phenyl moiety adjacent the·[Mn(CO)$_3$Br] metalation site and SIMU/RIGU restraints were used. To subtract the contribution from the disordered solvent (THF; note, one THF molecule was located, inserted as a fragment[3] and refined), a solvent mask was calculated, and 310 electrons were found in a volume of 1158 Å^3 in 1 void per unit cell.[1] This is consistent with the presence of an additional 2[C4H8O] per Asymmetric Unit which account for 320 electrons per unit cell. This reduction in electron density in the pore is consistent with loss of CO from the·[Mn(CO)$_3$Br] moiety and THF migration from the pores to the coordination site (where it is modelled).

MnMOF-1·[Mn(CO)$_3$Br] in THF (50 min of visible photolysis). Additional photolysis led to disorder of the [Mn(CO)$_3$Br] metalation site. This disorder was accommodated by modelling two distinct Mn(I) centres, Mn3 (the original·[Mn(CO)$_3$Br] metalation site, 0.2 site occupancy factor) and Mn3B (0.15 site occupancy factor). The EADP command was used to constrain the anisotropic displacement

parameter of Mn3B to be equal to that of Mn3. SIMU and RIGU restraints were used to model the coordinated axial CO ligand (ca. 0.075 occupancy now due to photolysis; note, there is no equatorial CO ligands) and DFIX restraints were used to enforce chemically reasonable bond lengths in the coordination sphere of the Mn atoms. The original coordinated Br site has dropped in occupancy further to a site occupancy factor of 0.16 due to scrambling across the axial sites (0.1 site occupancy factor at the second Mn centre), with the occupancy of the non-coordinated site holding steady at 0.09. The coordinated THF was modelled with a 0.7 site occupancy factor (i.e. coordinating both Mn centres in their equatorial sites) and SIMU, RIGU and DFIX restraints were used to give a chemically sensible structure. Disorder in the capping ligand had disappeared through the heating and cooling cycles but remained for the chelating site; a disorder model was developed, and a FLAT command was used to enforce planarity of the phenyl ring in the minor component of the disorder model. SIMU, RIGU and EADP restraints were further used to help refinement of this component. To subtract the contribution from the disordered solvent (THF; one THF molecule was located and refined with the aid of SIMU/RIGU restraints), a solvent mask was calculated, and 286 electrons were found in a volume of 1116 Å^3 in 1 void per unit cell.[1] This is consistent with the presence of an additional 1.75[C4H8O] per Asymmetric Unit which account for 280 electrons per unit cell. This reduction in electron density in the pore is consistent with further loss of CO from the·[Mn(CO)$_3$Br] moiety and THF migration from the pores to the coordination site (where it is modelled).

MnMOF-1·[Mn(CO)$_3$Br] in THF (90 min of visible photolysis). Disorder of the Mn(I) coordination sphere that emerged after 50 mins of irradiation was still present and was accommodated by modelling two distinct Mn centres, Mn3 and Mn3B. The occupancy of each metal centre was allowed to refine, and subsequently fixed at a site occupancy factor of 0.175 for each position. There was no evidence for residual CO in either the equatorial or axial positions (the Mn disorder and the bromine atoms in the axial sites obscured identification of the remaining ligands). A coordinated THF was inserted as a fragment from the Idealized Molecular Geometry Library.[3] DFIX restraints were used to enforce chemically reasonable bond lengths in the coordination sphere of the Mn atoms, including for the coordinated pyrazole. The original coordinated Br site has dropped in occupancy further to a site occupancy factor of 0.11 due to scrambling across the axial sites (0.165 site occupancy factor at the second Mn centre), with the occupancy of the non-coordinated site similar at a site occupancy factor 0.075. A FLAT command, along with SIMU and RIGU restraints, was used to enforce planarity and sensible thermal ellipsoids for the phenyl ring appended to the N,N-chelation site (the ligand is disordered but the disorder around the chelating site cannot be modelled). To subtract the contribution from the disordered solvent (THF; one THF molecule was located and refined with the aid of SIMU and RIGU restraints), a solvent mask was calculated and 280 electrons were found in a volume of 1134 Å^3 in 1 void per unit cell.[1] This is consistent with the presence of an additional 1.75[C4H8O] per Asymmetric Unit which account for 280 electrons per unit cell (i.e. no change from the previous structure).

MnMOF-1·[Mn(CO)$_3$Br] in THF (120 min of visible photolysis). Disorder of the Mn(I) coordination sphere that emerged after 50 mins of irradiation was still present and was accommodated by modelling two distinct Mn centres, Mn3 and Mn3B. The occupancy of each metal centre was allowed to refine, and subsequently fixed at site occupancy factors of 0.15 and 0.20 for Mn3 and Mn3B (a gradual increase in the occupancy of Mn3B). Aside from the Br anions, the only ligand included in the refinement was a THF molecule that was inserted as a fragment from the Idealized Molecular Geometry Library.[3] DFIX restraints were used to enforce chemically reasonable bond lengths in the coordination sphere of the Mn centre, including for the coordinated pyrazole. The original coordinated Br site has a site occupancy factor of 0.11 (0.2 site occupancy factor at the second Mn centre), with the occupancy of the non-coordinated site similar at a site occupancy factor

0.075. A FLAT command, along with SIMU and RIGU restraints, was used to enforce planarity and sensible thermal ellipsoids for the phenyl ring (the L'/bridging ligand is disordered but the disorder around the chelating site cannot be modelled). To subtract the contribution from the disordered solvent (THF; one THF molecule was located and refined with the aid of SIMU and RIGU restraints), a solvent mask was calculated and 292 electrons were found in a volume of 1182 Å^3 in 2 voids per unit cell.[1] This is consistent with the presence of an additional 1.75[C4H8O] per Asymmetric Unit which account for 280 electrons per unit cell (i.e. no major change from the previous structure).

MnMOF-1·[Mn(CO)$_3$MeCN]Br (initial). To subtract the contribution from the disordered solvent (MeCN), the SQUEEZE routine of PLATON was applied to the data, which generated a new HKL file. The number of located electrons was 335, which equates to approximately 15 acetonitrile molecules per unit cell, and 3.75 per asymmetric unit.[4] The occupancy of the bromide anion in MOF pore was fixed at 75% to fit with the electron density map (give the occupancy is confirmed as 100% there is likely a small contribution bound to the axial site of the Mn centre or at another site in the pore). DFIX, SIMU, and RIGU restraints were used in the refinement of carbonyl and acetonitrile ligands and the disordered phenyl rings in the MOF ligands (74 restraints in total).

MnMOF-1·[Mn(CO)$_3$MeCN]Br (10 min of UV photolysis). To subtract the contribution from the disordered solvent (MeCN), the SQUEEZE routine of PLATON was applied to the data, which generated a new HKL file.[4] The number of located electrons was 324, which equates to approximately 15 acetonitrile molecules per unit cell, and 3.75 per asymmetric unit. The occupancy of the bromide anion in MOF pore was again fixed at 75% to fit with the electron density map (see previous note). The equatorial ligands were modelled as 75% acetonitrile and 25% carbonyl. A series of DFIX, DANG, ISOR, SIMU and RIGU restraints were used in the refinement of carbonyl and acetonitrile ligands and the disordered phenyl rings in the MOF ligands (165 restraints in total).

MnMOF-1·[Mn(CO)$_3$MeCN]Br (20 min of UV photolysis). To subtract the contribution from the disordered solvent (MeCN), the SQUEEZE routine of PLATON was applied to the data, which generated a new HKL file.[4] The number of located electrons was 344, which equates to approximately 16 acetonitrile molecules per unit cell, and 4 per asymmetric unit. The occupancy of the coordinated bromine anion was fixed at 25%, while the uncoordinated bromine anion was fixed at 75% to best fit the electron density map. The occupancy of the axially coordinated carbonyl ligand was fixed at 25%. Attempts to increase this occupancy, or the occupancy of the bromine anion at this position lead to over occupation of the site (according to the electron density difference maps), and therefore it is assumed that the position is partially vacant. The equatorial and axial acetonitrile ligands were modelled at 100% occupancy. A series of DFIX, DANG, ISOR, SIMU and RIGU restraints were used in the refinement of bromide, carbonyl and acetonitrile ligands (125 restraints in total).

4.4 Thermal ellipsoid plots of the asymmetric units for all structures

4.4.1 MnMOF-1·[Mn(CO)$_3$Br]·THF photocrystallography experiment

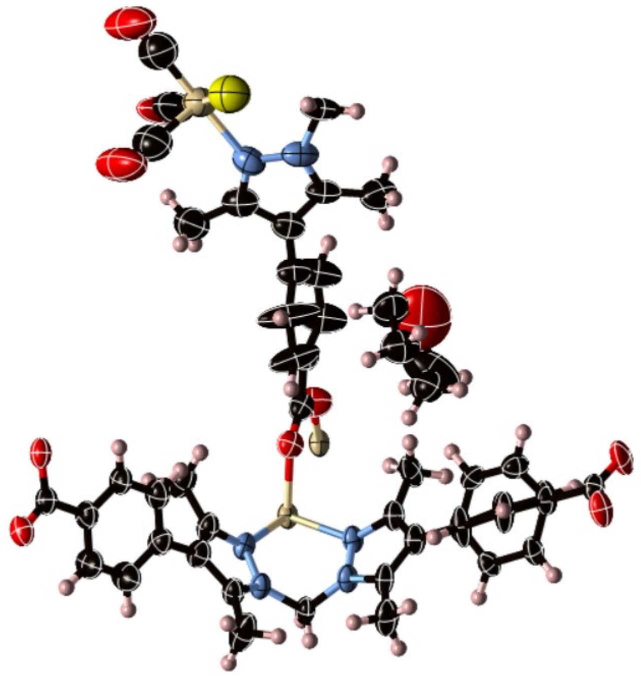

Figure S12. *The asymmetric unit of MnMOF-1·[Mn(CO)$_3$Br] with all non-hydrogen atoms represented by ellipsoids at the 50% probability level (C, black; H, white; N, aqua; O, red; Mn, beige; Br, yellow).*

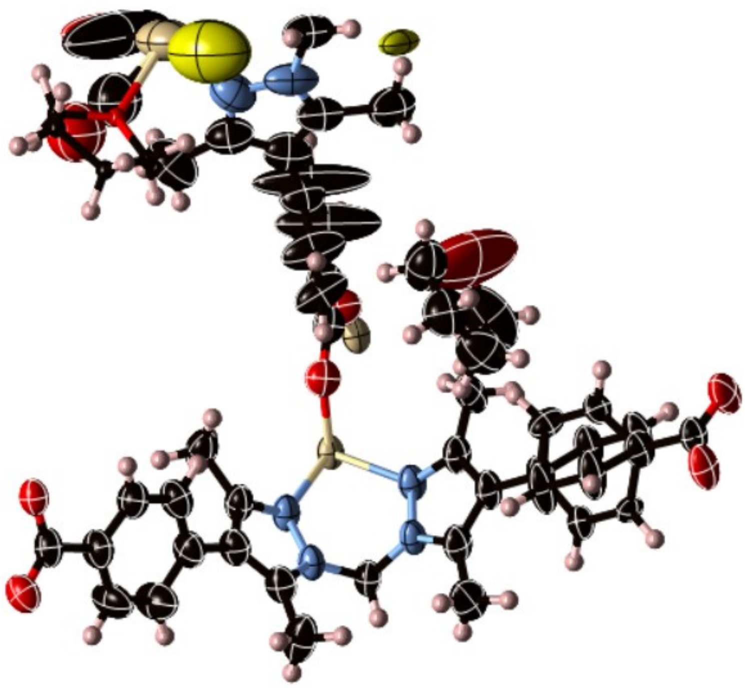

a)

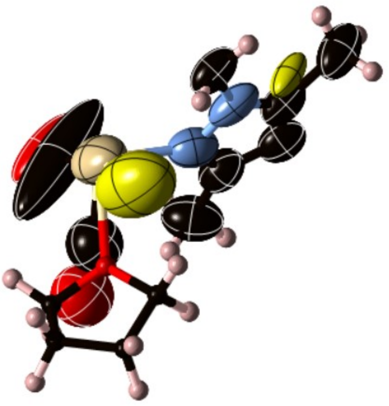

b)

Figure S13. *a) The asymmetric unit of unit of MnMOF-1·[Mn(CO)$_3$Br] after 30 minutes of photolysis in THF. All non-hydrogen atoms are represented by ellipsoids at the 50% probability level. (C, black; H, white; N, aqua; O, red; Mn, beige; Br, yellow). b) A perspective view of the Mn(I) coordination sphere with the non-hydrogen atoms represented by thermal ellipsoids at the 50% probability level.*

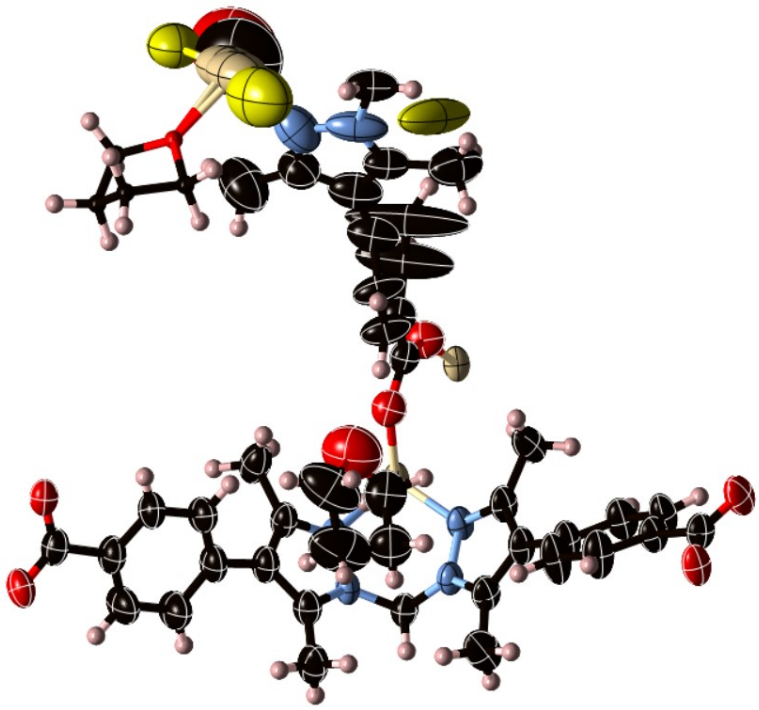

a)

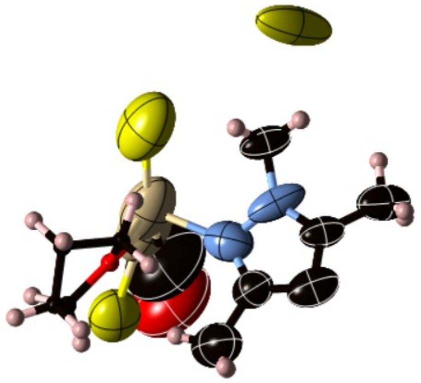

b)

Figure S14. *a) The asymmetric unit of unit of MnMOF-1·[Mn(CO)$_3$Br] after 50 minutes of photolysis in THF. All non-hydrogen atoms are represented by ellipsoids at the 50% probability level (C, black; H, white; N, aqua; O, red; Mn, beige; Br, yellow). b) A perspective view of the Mn(I) coordination sphere with the non-hydrogen atoms represented by thermal ellipsoids at the 50% probability level.*

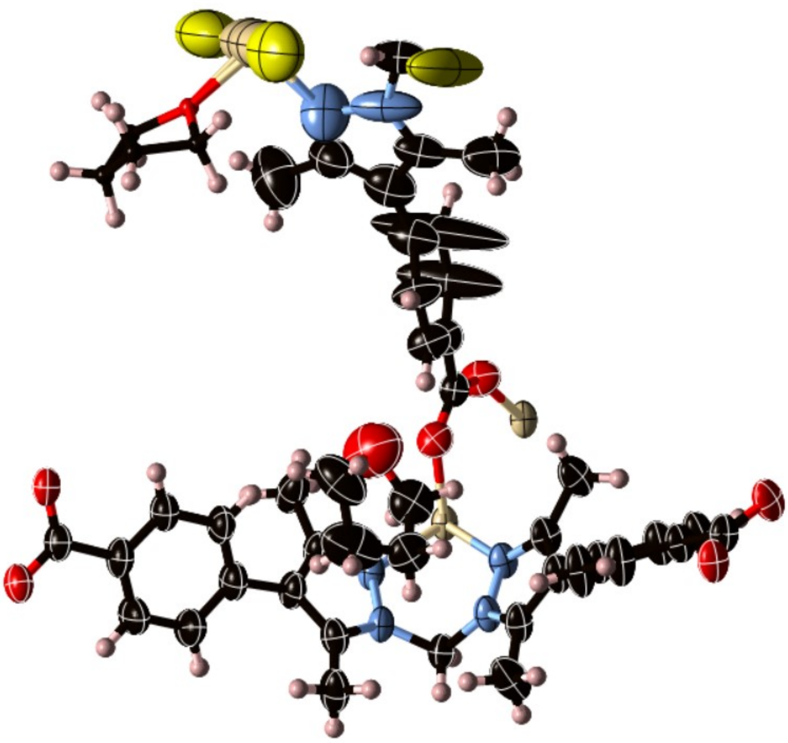

a)

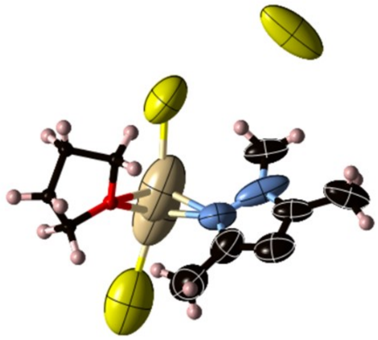

b)

Figure S15. *a) The asymmetric unit of unit of MnMOF-1·[Mn(CO)$_3$Br] after 90 minutes of photolysis in THF. All non-hydrogen atoms are represented by ellipsoids at the 50% probability level (C, black; H, white; N, aqua; O, red; Mn, beige; Br, yellow). b) A perspective view of the Mn(I) coordination sphere with the non-hydrogen atoms represented by thermal ellipsoids at the 50% probability level.*

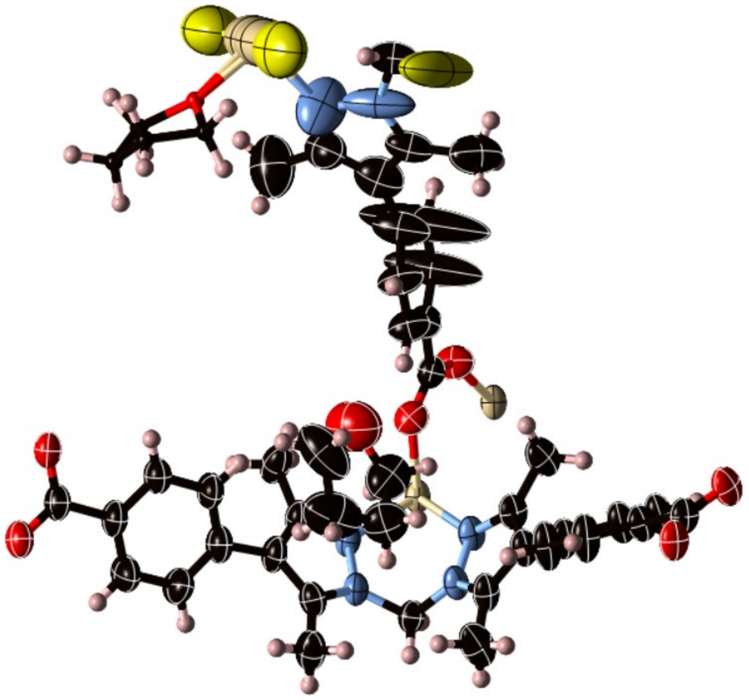

a)

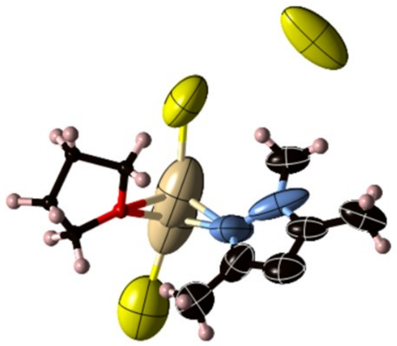

b)

Figure S16. *a) The asymmetric unit of unit of MnMOF-1·[Mn(CO)*$_3$*Br] after 120 minutes of photolysis in THF. All non-hydrogen atoms are represented by ellipsoids at the 50% probability level (C, black; H, white; N, aqua; O, red; Mn, beige; Br, yellow). b) A perspective view of the Mn(I) coordination sphere with the non-hydrogen atoms represented by thermal ellipsoids at the 50% probability level.*

4.4.2 MnMOF-1·[$Mn(CO)_3$MeCN]Br photocrystallography experiment

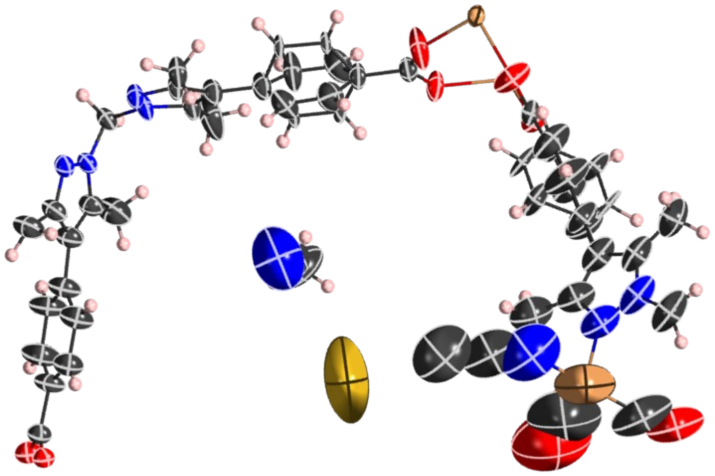

Figure S2. *Asymmetric unit of MnMOF-1·[$Mn(CO)_3$MeCN]Br, with all non-hydrogen atoms represented by ellipsoids at the 50% probability level. (C, grey; Br, yellow; Mn, beige; O, red; N, blue; H, pink).*

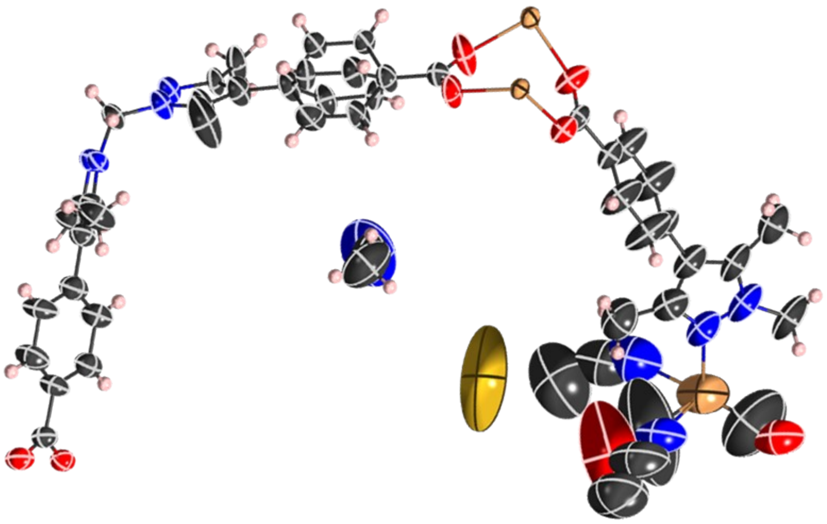

Figure S3. *Asymmetric unit of MnMOF-1·[$Mn(CO)_3$MeCN]Br photolyzed for 10 min, with all non-hydrogen atoms represented by ellipsoids at the 50% probability level. (C, grey; Br, yellow; Mn, beige; O, red; N, blue; H, pink).*

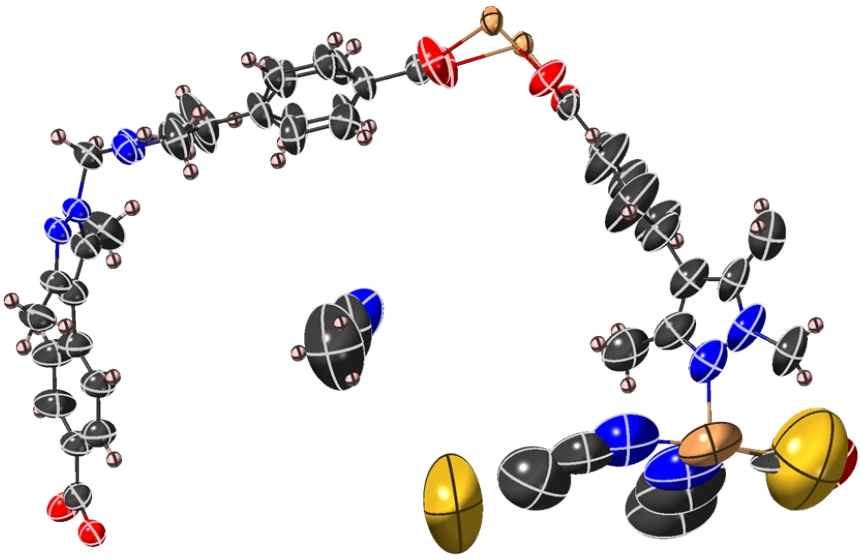

Figure S4. *Asymmetric unit of MnMOF-1·[Mn(CO)$_3$MeCN]Br photolyzed for 20 min, with all non-hydrogen atoms represented by ellipsoids at the 50% probability level. (C, grey; Br, yellow; Mn, beige; O, red; N, blue; H, pink).*

S4.5. Tables of data collection and refinement parameters

Table S4. Data collection and refinement parameters for MnMOF-1·[Mn(CO)$_3$Br] THF pre- and post-photolysis.

**Sample**	**MnMOF-1·[Mn(CO)$_3$Br] THF**	**MnMOF-1·[Mn(CO)$_3$Br] (THF) (30 mins)**	**MnMOF-1·[Mn(CO)$_3$Br] (THF) (50 mins)**
CCDC number	2219155	2219156	2219159
Formula	$C_{42.55}H_{39.8}Br_{0.35}Mn_{1.85}N_6O_{8.05}$	$C_{43.5}H_{43.8}Br_{0.35}Mn_{1.85}N_6O_{7.95}$	$C_{44.3}H_{46.6}Br_{0.35}Mn_{1.85}N_6O_{7.7}$
FW	893.61	907.45	915.88
T,K	100(2)	150(2)	150(2)
Wavelength, Å	Synchrotron (λ = 0.71073)	Synchrotron (λ = 0.71073)	Synchrotron (λ = 0.71073)
Crystal system, space group	Monoclinic, $P2_1/m$	Monoclinic, $P2_1/m$	Monoclinic, $P2_1/m$
Z	4	4	4
a, Å	12.337(3)	12.344(3)	12.338(3)
b, Å	32.664(7)	32.785(7)	32.702(7)
c, Å	12.964(3)	12.996(3)	13.010(3)
α˚	90	90	90
β, ˚	93.65(3)	93.93(3)	93.82(3)
γ˚	90	90	90
V, Å^3	5213.6(18)	5247.1(18)	5237.6(18)
d_{calc}, g/cm^3	1.138	1.149	1.161
Absorption coefficient, mm^{-1}	0.766	0.762	0.763
F(000)	1840.0	1876.0	1898.0
Crystal size, mm^3	0.18x0.03x0.01	0.18x0.03x0.01	0.18x0.03x0.01
Theta range for data collection	2.494 to 63.518	2.484 to 63.368	2.49 to 63.602
Index range	-17 ≤ h ≤ 17, -47 ≤ k ≤ 47, -17 ≤ l ≤ 17	-17 ≤ h ≤ 17, -48 ≤ k ≤ 48, -17 ≤ l ≤ 17	-17 ≤ h ≤ 17, -47 ≤ k ≤ 48, -17 ≤ l ≤ 17
Reflections collected	87994	88484	88776
Independent reflections	14332 [R_{int} = 0.0650, R_{sigma} = 0.0424]	14436 [R_{int} = 0.0677, R_{sigma} = 0.0452]	14470 [R_{int} = 0.0610, R_{sigma} = 0.0405]
Data/restraints/parameters	14332/86/597	14436/263/632	14470/241/626
Goodness-of-fit on F^2	1.039	1.289	1.240
Final R indexes [I>=2σ (I)]	R_1 = 0.1105, wR_2 = 0.3109	R_1 = 0.1136, wR_2 = 0.3302	R_1 = 0.1080, wR_2 = 0.3106
Final R indexes [all data]	R_1 = 0.1370, wR_2 = 0.3311	R_1 = 0.1506, wR_2 = 0.3554	R_1 = 0.1433, wR_2 = 0.3380
Largest diff. peak/hole / e Å^{-3}	1.64/-1.51	1.10/-1.36	2.15/-1.47

Table S5. Data collection and refinement parameters for MnMOF-1·[Mn(CO)$_3$Br] THF post-photolysis.

s	**MnMOF-1·[Mn(CO)$_3$Br] (THF) (90 mins)**	**MnMOF-1·[Mn(CO)$_3$Br] (THF) (120 mins)**
CCDC number	2219157	2219158
Formula	$C_{44.3}H_{43.6}Br_{0.35}Mn_{1.85}N_6O_{7.7}$	$C_{44.3}H_{46.6}Br_{0.35}Mn_{1.85}N_6O_{7.7}$
FW	912.86	915.88
T,K	100(2)	150(2)
Wavelength, Å	Synchrotron (λ = 0.71073)	Synchrotron (λ = 0.71073)
Crystal system, space group	Monoclinic, $P2_1/m$	Monoclinic, $P2_1/m$
Z	4	4
a, Å	12.338(3)	12.338(3)
b, Å	32.702(7)	32.702(7)
c, Å	13.010(3)	13.010(3)
α˚	90	90
β, ˚	93.82(3)	93.82(3)
γ˚	90	90
V, Å^3	5237.6(18)	5237.6(18)
d_{calc}, g/cm^3	1.158	1.161
Absorption coefficient, mm^{-1}	0.763	0.763
F(000)	1886.0	1898.0
Crystal size, mm^3	0.18x0.03x0.01	0.18x0.03x0.01
Theta range for data collection	2.49 to 54.998	2.49 to 63.682
Index range	-16 ≤ h ≤ 16, -42 ≤ k ≤ 42, -16 ≤ l ≤ 16	-17 ≤ h ≤ 17, -48 ≤ k ≤ 48, -17 ≤ l ≤ 17
Reflections collected	78054	88372
Independent reflections	12158 [R_{int} = 0.0546, R_{sigma} = 0.0315]	14456 [R_{int} = 0.0642, R_{sigma} = 0.0422]
Data/restraints/parameters	12158/201/580	14456/205/555
Goodness-of-fit on F^2	1.404	1.293
Final R indexes [I>=2σ (I)]	R_1 = 0.1121, wR_2 = 0.3294	R_1 = 0.1137, wR_2 = 0.3233
Final R indexes [all data]	R_1 = 0.1378, wR_2 = 0.3533	R_1 = 0.1519, wR_2 = 0.3543
Largest diff. peak/hole / e Å^{-3}	2.52/-1.39	2.61/-1.47

Table S6. Data collection and refinement parameters for MnMOF-1·[Mn(CO)$_3$MeCN] Br pre- and post-photolysis.

**Sample**	**MnMOF-1·[Mn(CO)$_3$MeCN]Br**	**MnMOF-1·[Mn(CO)$_3$MeCN]Br (10 mins)**	**MnMOF-1·[Mn(CO)$_3$MeCN]Br (20 mins)**
CCDC number	2219974	2219972	2219973
Empirical formula	$C_{42}H_{37.25}Br_{0.38}Mn_2N_{7.5}O_{7.5}$	$C_{42.75}H_{39.75}Br_{0.38}Mn_2N_{8.25}O_{6.75}$	$C_{42.75}H_{40.5}Br_{0.5}Mn_2N_{8.5}O_{6.25}$
Formula weight	915.36	910.52	923.17
Temperature/K	100(2)	100(2)	100(2)
Radiation	Synchrotron (λ = 0.6889)	Synchrotron (λ = 0.6889)	Synchrotron (λ = 0.6889)
Crystal system	monoclinic	monoclinic	monoclinic
Space group	$P2_1/m$	$P2_1/m$	$P2_1/m$
a/Å	12.4134(13)	12.4072(8)	12.4279(11)
b/Å	33.362(4)	33.106(2)	33.146(3)
c/Å	12.9953(13)	12.9591(9)	12.9816(13)
α/°	90	90	90
β/°	95.565(9)	95.378(6)	95.071(8)
γ/°	90	90	90
Volume/Å^3	5356.4(10)	5299.5(6)	5326.6(9)
Z	4	4	4
ρ_{calc}g/cm^3	1.135	1.141	1.151
μ/mm^{-1}	0.821	0.747	0.825
F(000)	1871.0	1858.0	1896.0
Crystal size/mm^3	0.1 × 0.03 × 0.005	0.1 × 0.03 × 0.005	0.1 × 0.03 × 0.005
2Θ range for data collection/°	2.366 to 71.67	2.384 to 71.768	2.382 to 71.712
Index ranges	-20 ≤ h ≤ 15, -53 ≤ k ≤ 56, -21 ≤ l ≤ 22	-20 ≤ h ≤ 15, -53 ≤ k ≤ 56, -21 ≤ l ≤ 20	-20 ≤ h ≤ 15, -53 ≤ k ≤ 56, -21 ≤ l ≤ 20
Reflections collected	81242	80877	80094
Independent reflections	24595 [R_{int} = 0.1626, R_{sigma} = 0.3312]	24448 [R_{int} = 0.1428, R_{sigma} = 0.3044]	24499 [R_{int} = 0.1406, R_{sigma} = 0.4545]
Data/restraints/parameters	24595/74/619	24448/165/621	24499/125/603
Goodness-of-fit on F^2	0.878	0.914	0.907
Final R indexes [I>=2σ (I)]	R_1 = 0.1209, wR_2 = 0.3187	R_1 = 0.1194, wR_2 = 0.3122	R_1 = 0.1303, wR_2 = 0.3507
Final R indexes [all data]	R_1 = 0.2576, wR_2 = 0.3530	R_1 = 0.2859, wR_2 = 0.3588	R_1 = 0.3346, wR_2 = 0.3995
Largest diff. peak/hole / e Å^{-3}	1.07/-0.99	1.17/-1.06	1.02/-1.07

S5. Calculations used to determine Co occupancy

Co occupancy was calculated by the following formula

c = #Co atoms

m = #Mn atoms

t= total number of Mn and Co atoms

$r = \frac{c}{m}$ (measured atomic ratio of Co to Mn)

$\therefore c = rm$

$t = c + m$

$= rm + m$

$= (r + 1)m$

Proportion of sites occupied $= \frac{c + m}{4}$ (as there are 4 potential sites for metal atoms to occupy)

$= \frac{(r + 1)m}{4}$

Co occupancy $= \frac{c}{Proportion\ of\ sites\ occupied}$

$= \frac{4c}{(r + 1)m}$ (as c=rm)

$= \frac{4rm}{(r + 1)m}$

Therefore, Co occupancy $= \frac{4r}{r + 1}$

S6. X-ray Photoelectron Spectroscopy (XPS) Details

As there are no previous reports of XPS spectra for Mn(I) species it was imperative to generate reference spectra for both Mn(I) and Mn(II) oxidation states. MnMOF-1·[Mn(CO)$_3$X]Y contains both, Mn(II) and Mn(I) cations with the Mn(II) cations, which act as nodes, being three times more abundant than the tethered Mn(I). Thus, the non-metallated MnMOF-1 and the previously reported model complex,[5] Mn(dpzm)(CO)$_3$Br (where dpzm = 2,2'-dipyrazinylmethane), were chosen as references for Mn(II) and Mn(I) respectively. For the light sensitive MnMOF-1·[Mn(CO)$_3$X]Y samples, all preparations were carried out in the dark and all openings on the reaction apparatus were covered with aluminium foil. Both the THF solvated and MeCN solvated MnMOF-1·[Mn(CO)$_3$X]Y samples were analysed, with XPS spectra collected from the unphotolysed samples before the samples were irradiated with a visible-light torch through a window in the reaction apparatus. Unwanted decarbonylation of the complex from the high vacuum and X-ray beam prior to photolysis was a concern, but an immediate increase in the chamber pressure (from 1 x 10^{-9} to 2.5 x 10^{-8} mbar) was observed when the light was turned on, indicating that CO was released from the MOF only under irradiation. This confirmed that the initial spectra collected for each sample arose from the parent complex, and the second spectrum was for the fully photolyzed product, as there was no means to perform *in situ* IR spectroscopy. The samples were photolyzed until the chamber pressure returned to pre-photolysis levels, indicating that all CO had been removed. Although the C-O peak in the C 1s region could be observed in the Mn(I) reference, it could not be seen in the MOF samples due to the confounding presence of the other C-X bonds. Therefore, only the Mn regions of the spectrum were of interest in comparing the pre- and post-photolysis samples. A Shirley background was used as a linear background did not improve the fitting parameters.

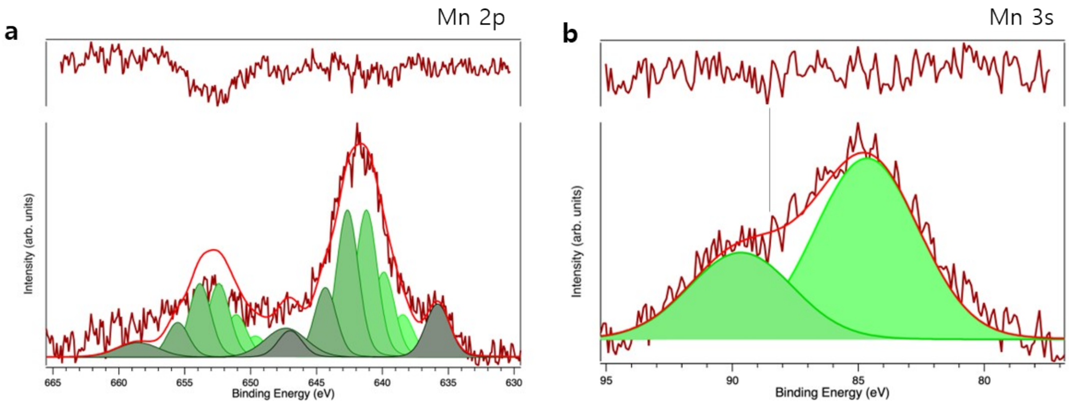

Figure 20. *XPS spectra of the Mn(I) model sample [Mn(dpzm)(CO)$_3$Br]. a) The Mn 2p region and* ***b)*** *Mn 3s regions.*

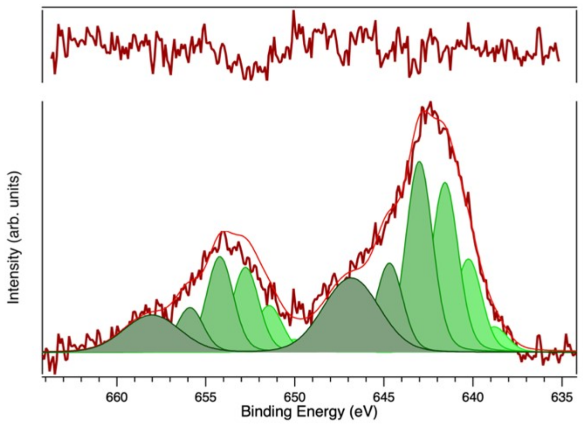

Figure 21. *XPS spectra of non-metallated MnMOF-1 for the Mn 2p region.*

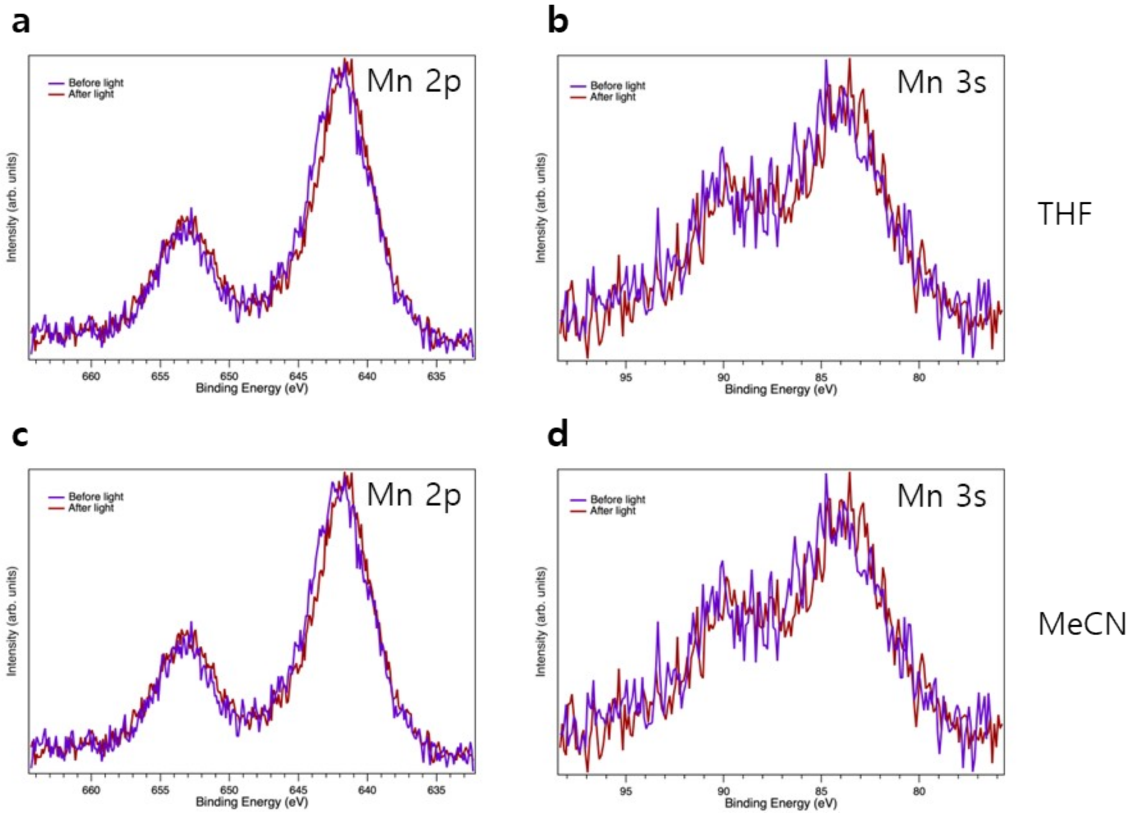

Figure 22. *XPS spectra of MnMOF-1·[Mn(CO)$_3$X]Y samples before (purple) and after (red) photolysis of the sample dried from THF a) Mn 2p and b) Mn 3s regions; and the sample dried from MeCN c) Mn 2p and d) Mn 3s regions.*

S7. References

1 P. van der Sluis and A. L. Spek, *Acta Cryst*., 1990, **A46**, 194-201.

2 W. M. Bloch, A. Burgun, C. J. Coghlan, R. Lee, M. L. Coote, C. J. Doonan and C. J. Sumby, *Nat. Chem.*, 2014, **6**, 906–912.

3 I. A. Guzei, *J. Appl. Crystallogr.*, 2014, **47**, 806-809.

4 A. L. Spek, *Acta Cryst.*, 2015, **C71**, 9–18.

5 M. T. Huxley, A. Burgun, H. Ghodrati, C. J. Coghlan, A. Lemieux, N. R. Champness, D. M. Huang, C. J. Doonan and C. J. Sumby, *J. Am. Chem. Soc.*, 2018, **140**, 6416–6425.
